# Biflavonoids from *Selaginella doederleinii* as Potential Antitumor Agents for Intervention of Non-Small Cell Lung Cancer

**DOI:** 10.3390/molecules26175401

**Published:** 2021-09-05

**Authors:** Fenghua Kang, Sha Zhang, Dekun Chen, Jianbing Tan, Min Kuang, Jinlin Zhang, Guangyuan Zeng, Kangping Xu, Zhenxing Zou, Guishan Tan

**Affiliations:** 1 Xiangya School of Pharmaceutical Sciences, Central South University, Changsha 410013, China; kangfenghua@csu.edu.cn (F.K.); ad8879789@163.com (S.Z.); 197211019@csu.edu.cn (D.C.); tanjb1009@csu.edu.cn (J.T.); kuangmin@csu.edu.cn (M.K.); Jeannie_z@163.com (J.Z.); 207211012@csu.edu.cn (G.Z.); xukp395@csu.edu.cn (K.X.); 2 Xiangya Hospital of Central South University, Changsha 410008, China; 3 Hunan Key laboratory of Diagnostic and Therapeutic Drug Research for Chronic Diseases, Central South University, Changsha 410013, China

**Keywords:** *Selaginella doederleinii*, biflavonoid, antiproliferative activity, apoptosis, cell cycle

## Abstract

Four new biflavonoids (**1**–**4**) were isolated from *Selaginella doederleinii* together with a known biflavonoid derivative (**5**). Their structures contained a rare linker of individual flavones to each other by direct C-3-O-C-4′′′ bonds, and were elucidated by extensive spectroscopic data, including HRESIMS, NMR and ECD data. All isolates significantly inhibited the proliferation of NSCLC cells (IC_50_ = 2.3–8.4 μM) with low toxicity to non-cancer MRC-5 cells, superior to the clinically used drug DDP. Furthermore, the most active compound **3** suppressed XIAP and survivin expression, promoted upregulation of caspase-3/cleaved-caspase-3, as well as induced cell apoptosis and cycle arrest in A549 cells. Together, our findings suggest that **3** may be worth studying further for intervention of NSCLC.

## 1. Introduction

Non-small cell lung cancer (NSCLC) accounts for 85% of all lung cancer cases, and remains the principal cause for cancer-related death worldwide [1,2]. The 5-year relative survival rate of NSCLC patients is less than 15% [3]. Therefore, novel therapeutic agents including safe and effective anti-NSCLC compounds are urgently needed.

It is well known that diverse bioactive secondary metabolites derived from plants provide a natural source of novel agents with distinct pharmaceutical activities. Natural biflavonoids are known to link the individual flavones to each other by direct C-C or C-O-C bonds, forming dimeric structures capable of having multiple therapeutic benefits [4,5]. Meanwhile, it has been reported that natural bioflavonoids have promising therapeutic effects on metabolic-, cardiovascular-, and cognition-related diseases, including cancer [5].

*Selaginella*, belonging to the plant family *Selaginellaceae*, is known to be a rich source of bioflavonoids [6,7,8,9], and is frequently used as a Traditional Chinese Medicine for the treatment of malignant tumors [10]. Until now, approximately 80 biflavonoids have been reported from *Selaginella* genus, some of which can act as a potential anti-cancer molecule involving multiple factors, including the induction of apoptosis, retardation of the angiogenic and metastatic cascades, etc. [11,12]. Interestingly, in recent years, we have studied the secondary metabolites of the *Selaginella* genus, seeking the characteristic chemical composition of the plants for medicinal use of *Selaginella*, and a series of new bioactive flavonoids have been found [13,14,15,16,17,18]. Notably, the chemical investigation reported previously by our research group on *Selaginella doederleinii*, resulted in the isolation of several new biflavones, which significantly inhibited the proliferation of NSCLC cells [13,19].

**Figure 1:**
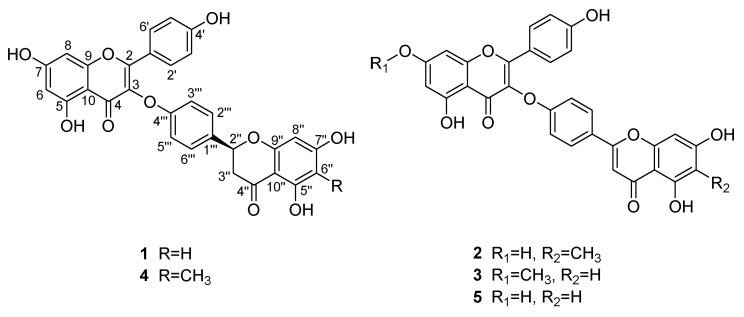
Structures of compounds **1**–**5**.

In our continuing search, the active ingredients of the air-dried whole herbs of *S. doederleinii* were further investigated. Four previously undescribed biflavonoids (**1**–**4**), and a known biflavonoid derivative compound, delicaflavone (**5**) [20] were isolated from 75% EtOH extracts. Interestingly, all isolated compounds represented one of the rare biflavonoids family bearing a linker of individual flavones to each other by direct C-3-O-C-4′′′ bonds. Accordingly, the isolation and structural elucidation of compounds **1**–**5** (Figure 1), as well as their antiproliferative activity against NSCLC cells, potentiated apoptosis-mediated death of A549 cells and affected cell cycle arresting were described.

## 2. Results

### 2.1. Structural Elucidation

Compound **1** was obtained as yellow amorphous powder. Its molecular formula was established to be C_30_H_20_O_10_ on the basis of a molecular ion peak at *m/z* 541.1127 [M + H]^+^ (calcd. for C_30_H_21_O_10_, 541.1129) in its HRESIMS spectrum. The ^1^H NMR spectrum of **1** (Table 1) exhibited signals for twelve aromatic protons, corresponding to two groups A_2_B_2_ coupled protons at *δ*_H_ 7.45 (2H, d, *J* = 8.5 Hz, H-2′′′, 6′′′), 7.10 (2H, d, *J* = 8.5 Hz, H-3′′′, 5′′′), 7.87 (2H, d, *J* = 8.5 Hz, H-2′, 6′), and 6.89 (2H, d, *J* = 8.5 Hz, H-3′, 5′); two sets of meta-coupled protons at *δ*_H_ 6.54 (1H, d, *J* = 2.0 Hz, H-8), 6.24 (1H, d, *J* = 2.0 Hz, H-6), 5.90 (1H, d, *J* = 2.0 Hz, H-8″), and 5.89 (1H, *J* = 2.0 Hz, H-6″), respectively. In addition, two methylene protons at *δ*_H_ 3.26 (1H, dd, *J* = 17.0, 3.0 Hz, H-3″) and 2.71 (1H, dd, *J* = 17.0, 3.0 Hz, H-3″); one oxygenated methine proton at *δ*_H_ 5.50 (1H, dd, *J* = 13.0, 3.0 Hz, H-2″), and five exchangeable protons at *δ*_H_ 12.30 (1H, s, 5-OH), 12.14 (1H, s, 5″-OH), 10.96 (1H, br s, 7-OH), 10.82 (1H, br s, 7″-OH), and 10.37 (1H, br s, 4′-OH). The ^13^C NMR spectrum (Table 2) showed signals for 30 carbons, including two carbonyl groups, twenty-four aromatic carbons, two olefinic carbons, one methylene carbon, and one oxymethine carbon.

**Figure 2:**
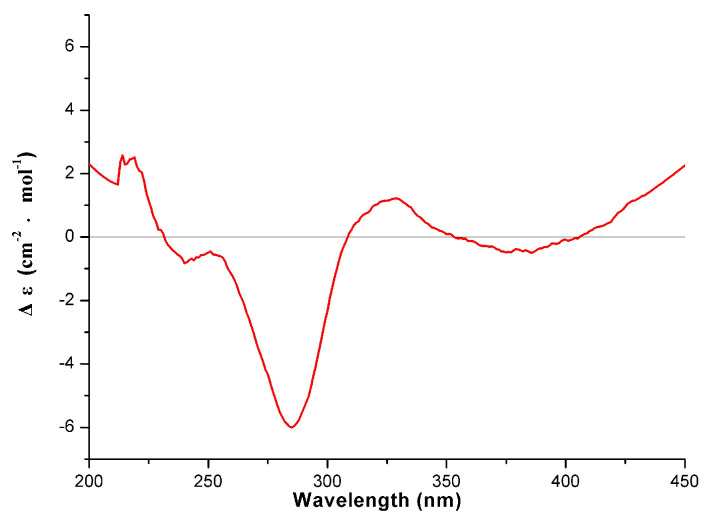
CD spectrum of compound **1** (in MeOH).

Detailed analysis of the 1D and 2D NMR data (Table 1 and Table 2) from compound **1** indicated that it was a diflavonoid composed of two flavanone units, likewise. Comparisons to naringeninyl-(4′′′,*O*,3)-kaempferol [21], previously isolated from the *Selaginella chrysocaulos*, revealed that the two compounds share same planar structure, however, when reported in racemic mixture form in the literature, no absolute configuration was elaborated. The CD spectrum (Figure 2) of **1** showed a positive Cotton effect at 330 nm and a negative Cotton effect at 290 nm, indicating an *S* configuration [22]. Based on the above analysis, the structure of **1** was elucidated as (2″*S*)-2″,3″-dihydrodelicaflavone.

**Figure 3:**
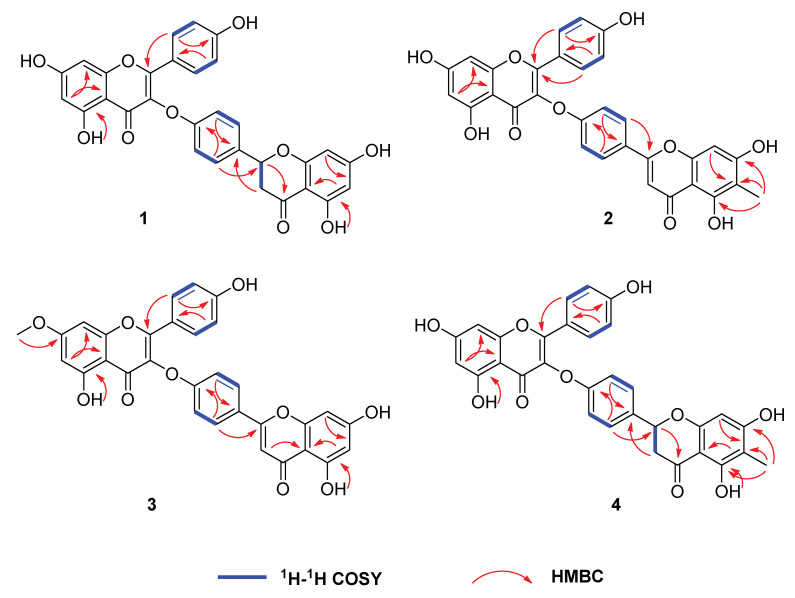
Key ^1^H-^1^H COSY and HMBC correlations of compounds **1**–**4**.

Compound **2** was obtained as a yellow amorphous powder. Its molecular formula was determined to be C_31_H_20_O_10_ based on its HRESIMS data at *m*/*z* 553.1128 [M + H]^+^ (calcd. for C_31_H_21_O_10_, 553.1135). The NMR data (Table 1 and Table 2) of **2** also showed high similarity to those of delicaflavone (**5**), with the only noticeable difference in the NMR data and spectra being the presence of an additional methyl signal at C-6″. This deduction was further verified by the HMBC correlations from 6″-CH_3_ (*δ*_H_ 1.97) to C-5″ (*δ*_C_ 158.8), C-6″ (*δ*_C_ 107.8), and C-7″ (*δ*_C_ 165.4) (Figure 3). Hence, the structure of **2** was determined as 6″-methyldelicaflavone.

Compound **3** was obtained as a yellow amorphous powder and its molecular formula was established as C_31_H_20_O_10_ by its HRESIMS *m/z* 553.1119 [M + H] ^+^ (calcd. for C_31_H_21_O_10_, 553.1129). Analysis of the above ^1^H NMR and ^13^C NMR data (Table 1 and Table 2) suggested that **3** should be a biflavonoid consisting of two flavanone units, likewise. Comparing the NMR data of **3** and delicaflavone (**5**), their main difference was that the hydroxyl group of one flavanone was replaced by a methoxyl group. The result can be confirmed by HMBC correlation of 7-OCH_3_ (*δ*_H_ 3.87) with C-7 (*δ*_C_ 164.7) (Figure 3). Therefore, the structure of **3** was deduced as 7-*O*-methyldelicaflavone.

**Figure 4:**
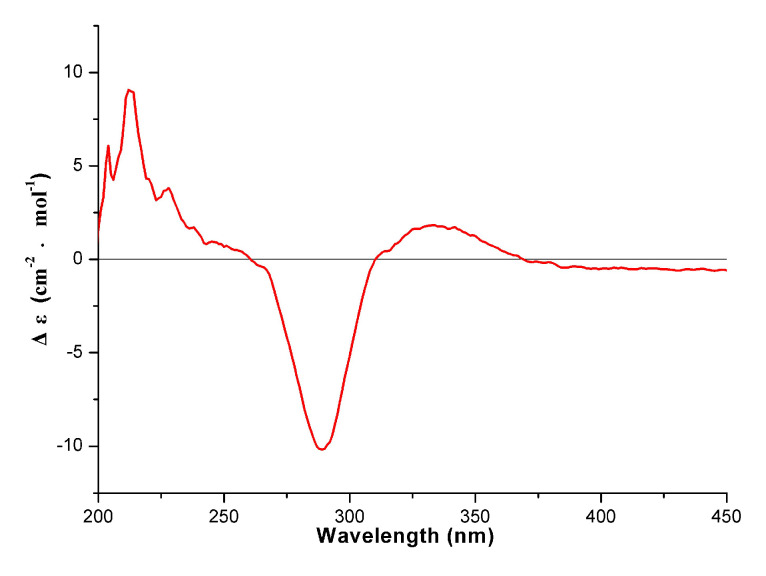
CD spectrum of compound **4** (in MeOH).

Compound **4** was obtained as a yellow amorphous powder. A molecular formula of C_31_H_22_O_10_ was determined from the molecular ion peak at *m*/*z* 555.1299 [M + H]^+^ (calcd. for C_31_H_23_O_10_, 555.1286) obtained by HRESIMS spectrum. The 1D and 2D NMR data (Table 1 and Table 2) were almost identical to **1** except for an additional methyl at C-6″ in **4**, which can be proved via the key HMBC correlations of 6″-CH_3_ (*δ*_H_ 1.88) with C-5″ (*δ*_C_ 161.2), C-6″ (*δ*_C_ 103.8), and C-7″ (*δ*_C_ 165.1) (Figure 3). The absolute configuration at C-2″ was determined to be *S* configuration by the presence of a negative Cotton effect at 290 nm and a positive Cotton effect at 330 nm in its CD spectrum (Figure 4). Consequently, compound **4** was characterized as (2″*S*)-6″-methyl-2″,3″-dihydrodelicaflavone.

### 2.2. Assessment of Anti-Proliferative Activity of Compounds ***1**–**5*** in NSCLC Cells

**Figure 5:**
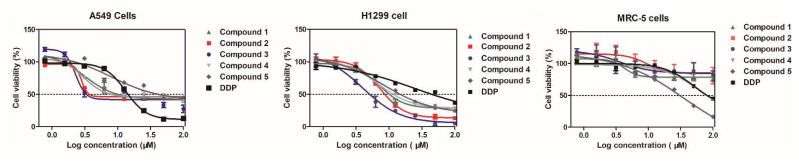
Antiproliferative activity of compounds **1**–**5** against two NSCLC cell lines (A549 and H1299) as well as non-cancer MRC-5 cells.

To evaluate the anti-NSCLC activity of compounds **1**–**5**, we tested their inhibitory effect on the proliferation of two NSCLC cell lines (A549 and H1299) as well as human normal embryonic lung cell line MRC-5 by the MTT assay using *Cis*-platin (DDP) as a positive control. As shown in Table 3 and Figure 5, all compounds exhibited potent inhibitory activity against both A549 (IC_50_ = 2.3–7.9 μM) and H1299 cells (IC_50_ = 4.0–8.4 μM), and their antiproliferative activity was more potent than DDP (IC_50_ = 13.3 and 24.5 μM, respectively). In contrast, there was no obvious toxicity of compounds **1**–**5** in non-cancer MRC-5 cells, suggesting that **1**–**5** preferentially and effectively inhibited the growth of NSCLC cells in vitro. Among them, compounds **1**–**3** were further studied for their potential apoptosis-mediated death of A549 cells because of their promising antiproliferative activity against NSCLC cells.

### 2.3. Compound ***1**–**3*** Induced Apoptosis in A549 Cells

**Figure 6:**
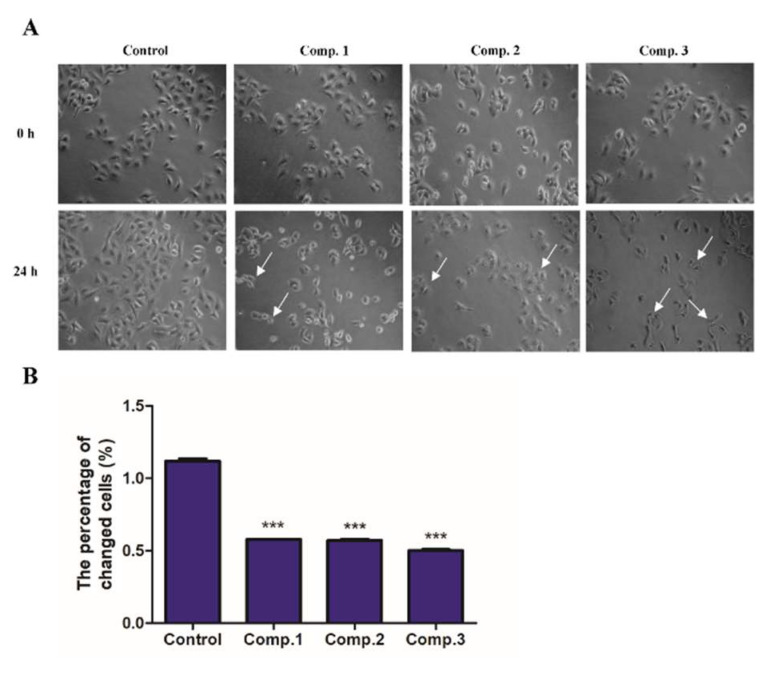
Morphological changes of A549 cells. (**A**) The cell lines were exposed to equimolar (3 μM) concentration of compounds **1**–**3**, following 24 h treatment, versus control group (untreated cells). Scale bars, 50 μm. White arrows show the apoptosis cells. (**B**) Quantitative analysis of live cells. Data are expressed as means from three independent experiments. *** *p* < 0.001 vs the 0 h group.

To investigate the effect of **1**–**3** on the induction of NSCLC cell apoptosis, a series of experiments were conducted in A549 cell lines. Initially, we determined the impact of **1**–**3** treatment on the morphological features of A549 cells using phase-contrast microscopy (Figure 6). It was observed that treatment with **1**–**3**, and especially with compound **3,** significantly reduced the viability of A549 cells, and morphological changes in A549 cells, such as shrinkage, detachment and reduction in the number of live cells, were indicative of apoptosis.

**Figure 7:**
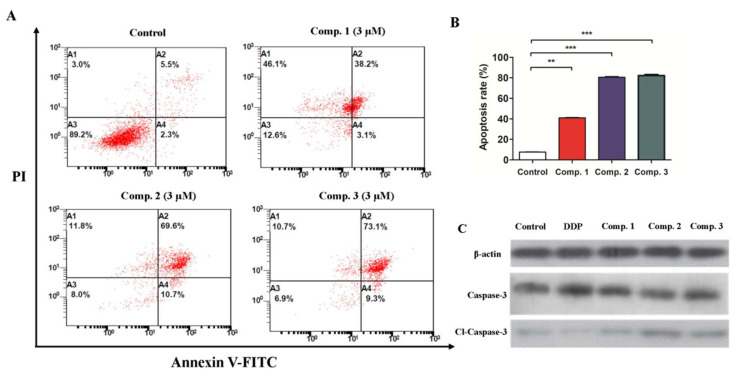
Compounds **1**–**3** induced A549 cell apoptosis. (**A**) The representational pictures of flow cytometry in cell apoptosis with or without indicated compound 3 μM for 24 h. (**B**) Apoptosis rates of compounds **1**–**3** as well as control group. (**C**) The expression of apoptosis related proteins after the treatment of compound **1**–**3** for 24 h by Western blot assay. Data are expressed as means of the percentages of apoptotic cells from three independent experiments. ** *p* < 0.01, *** *p* < 0.001 vs control group.

Subsequently, to further verify the apoptosis induction potential of compounds **1**–**3**, quantification of Annexin V-FITC/PI double positive cells was performed using flow cytometry analysis. A549 cells were incubated with indicated compounds, and the percentages of apoptotic A549 cells were determined by FITC-Annexin V and PI staining and flow cytometry. As shown in Figure 7A,B, compared with the control group, treatment with compounds **1**, **2** and **3** resulted in 41.3%, 80.3% and 82.4% apoptosis incidence in A549 cells, respectively. Collectively, these data clearly demonstrate that the inductive effect of **2** was significantly stronger than that of compound **1** but less than that of compound **3** at the same dose, and were quite well associated with the in vitro antiproliferative activity of compounds **1**–**3** in A549 cells.

### 2.4. Compound ***1**–**3*** Promoted Upregulation of Caspase-3/Cleaved-Caspase-3 in A549 Cells

Furthermore, to elucidate the mechanism underlying the apoptosis-inducing activity of compounds **1**–**3**, we examined their regulatory effect on the expression of caspase-3/cleaved-caspase-3, which are hallmarks of apoptosis. As shown in Figure 7C and Supplementary FigureS S1–S29, the Western blot assay revealed that compounds **1**–**3** increased the relative levels of caspase-3 and cleaved caspase-3 to different degrees in A549 cells. Similarly, treatment with DDP also enhanced caspase-3 and cleaved caspase-3, but the regulatory effect of DDP on cleaved-caspase-3 in A549 cell lines was less than that of compounds **2** and **3**. Together, these results indicated that compounds **1**–**3** may exert their antiproliferative activity, at least in part, via induction of the A549 cells’ apoptosis by caspase-3/cleaved-caspase-3 dependent mechanism.

### 2.5. Compound ***1**–**3*** Reduced the of Expression of XIAP and Survivin in A549 Cells

**Figure 8:**
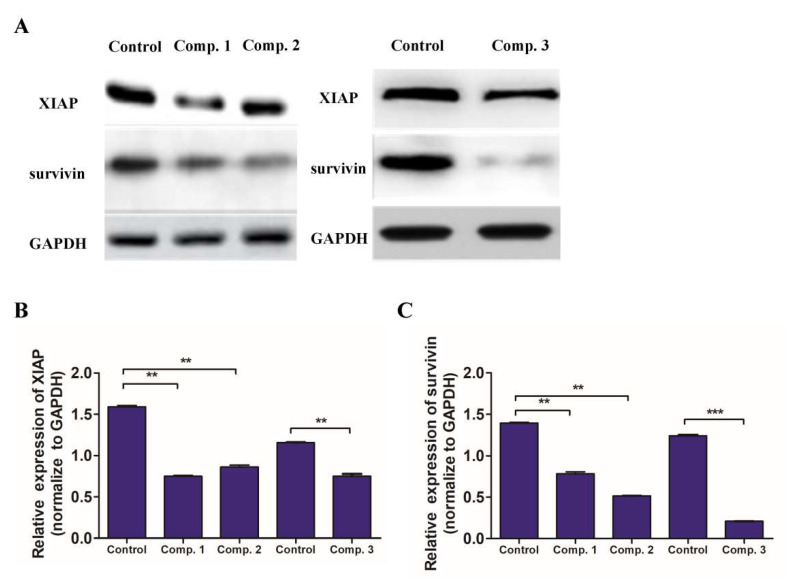
Effects of compounds **1**–**3** on expression of XIAP and survivin. A549 cells were treated with vehicle or the indicated compound for 24 h, and the relative levels of XIAP and survivin were determined by Western blot assays using GAPDH as a control. (**A**) Representative expression of XIAP and survivin. (**B**) Quantitative analysis of XIAP. (**C**) Quantitative analysis of survivin. Data are expressed as means from three independent experiments. ** *p* < 0.01, *** *p* < 0.001 vs control group.

The X-linked mammalian inhibitor of apoptosis protein (XIAP) and survivin, known as the inhibitor-of-apoptosis protein (IAP) members, play an important regulatory role in the caspase-dependent apoptosis [23]. Therefore, the Western blot assay experiments were further performed to examine whether the inhibition of XIAP and survivin were involved in compounds **1**–**3** induced apoptosis. As shown in Figure 8, all compounds significantly reduced the levels of XIAP and survivin expression, suggesting that compounds **1**–**3**, particularly compound **3,** may inhibit the XIAP and survivin expression, contributing to their apoptosis-inducing activity in NSCLC cells.

### 2.6. Compounds ***3*** Caused Cell Cycle Arrest in A549 Cells

**Figure 9:**
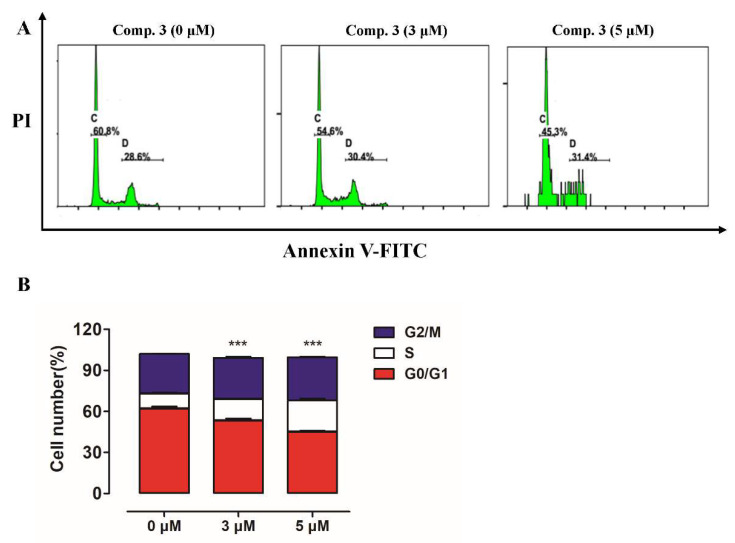
Cell cycle analysis of A549 cells after treatment with compound **3**. After 24 h of treatment, cells were labeled with PI (Propidium Iodide) and cell cycle analysis was performed using flow cytometry. (**A**) Representative histograms. (**B**) Quantitative analysis. Data are expressed as means from three independent experiments. *** *p* < 0.001 vs control group.

In addition to apoptosis, the induction of cell cycle arrest is an important means to control the growth and development of cancer cells. To investigate whether **3** suppressed the cells’ growth by arresting the cell cycle, we performed the experiment where cell cycle distribution was analyzed with flow cytometry after staining the DNA with propidium iodide (PI). It was found that treatment with **3** increased the percentage of cells at the S phase from 10.6% to 24.8% while decreasing G0/G1-phase cells from 60.8% to 45.3% (Figure 9). These results suggested that **3** may induce A549 cell cycle arrest at S phase.

## 3. Discussion

Natural biflavonoids, such as amentoflavone, bilobetin, ginkgetin, isoginkgetin, taiwaniaflavone, morelloflavone, delicaflavone and hinokiflavone isolated from *Selaginella* sp., *Ginkgo biloba*, *Garcinia* sp., and several other species of plants, are known to link the individual flavones to each other by direct C-C or C-O-C bonds [5]. During our continuous effort to discover active ingredients of the air-dried whole herbs of *S. doederleinii*, a series of dimeric flavones, including four previously undescribed biflavonoids, were isolated. Interestingly, these compounds represented one of a rare biflavonoids family bearing a linker of individual flavones to each other by direct C-3-O-C-4′′′ bonds, which was isolated for the first time from *Selaginella doederleinii*. 

The traditional roles of biflavones in cancer therapy can be defined with respect to the inhibition of metabolism-related processes/pathways, enzymes or proteins, especially for apoptosis induction mechanisms [5]. Therefore, it is pertinent to evaluate the effect of biflavones on different proteins or mechanisms that lead to cell apoptosis in cancer cells. In this study, we isolated these rare biflavonoids mentioned above from *S. doederleinii*, and further studied their anti-NSCLC effects and molecular mechanisms for the first time, to our knowledge. We demonstrated that compounds such as **3** inhibit proliferation, and induce apoptosis and cell cycle of NSCLC cells. Cell apoptosis induced by the caspases family is a regulated physiological process leading to cell death. Initiator caspases are closely coupled to multiple proapoptotic signals, such as XIAP and survivin signaling pathways [23]. Once activated, these caspases cleave and activate downstream effector caspases (mainly caspase3), which in turn cleave cytoskeletal and nuclear proteins, and finally induce apoptosis [24]. Thus, we conducted a series of experiments to investigate the effect of **1**–**3** on the induction of NSCLC cell apoptosis, and the mechanism underlying the apoptosis-inducing activity. We speculated that compounds **1**–**3** could induce apoptosis preliminarily through their in vitro antiproliferative activity and morphological observation. To further elaborate the fact that compounds **1**–**3** could induce cell apoptosis, Annexin V/PI staining assay was performed. The results showed that compared with the control group, treatment with compounds **1**, **2** and **3** resulted in 41.3%, 80.3% and 82.4% apoptosis incidence in A549 cells, respectively. Notably, these data on the apoptosis rate were quite well associated with the in vitro antiproliferative activity of compounds **1**–**3** in A549 cells. Furthermore, the Western blot assay revealed that compounds **1**–**3** suppressed the expression of anti-apoptotic proteins XIAP and survivin, but increased the relative levels of caspase-3/cleaved caspase-3 in A549 cells, suggesting that the reduced XIAP and survivin expression in compounds **1**–**3** treated cancer cells may lead to apoptosis via a caspase-3/cleaved-caspase-3 dependent mechanism. In addition, the results of cell cycle distribution after treatment with the most active compound **3** showed that **3** caused S arrest in A549 cells, suggesting that S arrest of the cell cycle may also contribute to the antiproliferative activity of **3**. Of course, the precise mechanisms underlying the action of **3** remain to be further investigated in the future.

In conclusion, the present study reports four new biflavonoids as well as delicaflavone (**5**), which were isolated from the whole herbs of *S. doederleinii*. The results of biological evaluation suggested that the anti-proliferative activity of the most active compound **3** could be attributed to the decreasing expression of anti-apoptotic proteins XIAP and survivin, leading to the activation of caspase-3/cleaved-caspase-3 and apoptosis of A549 cells. Additionally, cell cycle assays revealed that **3** arrested NSCLC cells at the S phase. Thereby, our findings may provide evidence that biflavonoids such as compound **3** act as potential antitumor agents, to be further investigated for NSCLC intervention. The potential therapeutic value of **3**, as well as its analogues, in NSCLC and other cancers warrants further study.

## 4. Materials and Methods

### 4.1. General Experimental Procedures

Optical rotations were measured on a JASCO P-1020 polarimeter (Horiba, Tokyo, Japan). HRESIMS data were recorded with a Finnigan LTQ-FT (Thermo Fisher, Waltham, MA, USA). CD spectra were obtained on an Applied Photophysics spectrometer (Chirascan, New Haven, CT, USA). The NMR spectra were recorded in DMSO-*d*_6_ with a Bruker AV-500/800 MHz spectrometer (Bruker, Karlsruhe, Germany) using tetramethylsilane (TMS) as the internal standard. Silica gel (200–300 or 60–100 mesh); Qingdao Peremanent Sea Silica Ltd., Qingdao, China), Sephadex LH-20 (TOYOPEARL TOSOH, Tokyo, Japan), and HW-40C (TOYOPEARL TOSOH, Tokyo, Japan) were used for column chromatography (CC). Semi-preparative HPLC was performed on an Agilent 1200 chromatograph equipped with DAD detector (Agilent Technologies, CA, USA) using a YMC Pack ODS-A RP-18 column (10 μm, 250 × 10 mm, YMC Co. Ltd., Kyoto, Japan). All solvents were analytical grade.

### 4.2. Plant Material

*S. doederleinii* samples were collected from the town of Wutong, Lingui district, Guangxi Zhuang Autonomous Region, China, during July 2013. The voucher specimen (20130710) was identified by Prof. Zhen-Ji Li (Xiamen University, Xiamen, China) and has been deposited in Xiangya School of Pharmaceutical Sciences, Central South University.

### 4.3. Extraction and Isolation

The air-dried whole herbs of *S. doederleinii* (10.0 kg) were extracted with 75% EtOH under reflux (2/60 L, 3 h/each) to get crude extracts. The alcoholic extract was evaporated under reduced pressure to give 1.1 kg of crude extract, which was then suspended in water and extracted with petroleum ether (PE), EtOAc and n-BuOH, respectively.

The EtOAc fraction (90 g) was subjected to silica gel CC eluted with mixture solvents CH_2_Cl_2_-MeOH-H_2_O (*v*/*v*/*v*, 100:0:0–0:50:50) to give 8 fractions (Fr.1–8). Fr.3 was separated on a HW-40C with MeOH-H_2_O (*v*/*v*, 60:40) to obtain 4 fractions (Fr.3–1 to Fr.3–4). Fr.3–2 was purified by silica gel CC eluting with PE-EtOAc (*v*/*v*, 30:0–0:100) to give 5 fractions (Fr.3-2-1 to Fr.3-2-5). Fr.3-2-3 was subjected to Sephadex LH-20 and eluted with MeOH to produce compound **1** (4.2 mg) and three fractions (Fr.3-2-3-1 to Fr.3-2-3-3). Fr.3-2-3-2 was further purified by semi-preparative HPLC (ACN/H_2_O, 40–60%) to afford compounds **3** (8.9 mg), **4** (3.7 mg). Fr.3-2-4 was further purified by semi-preparative HPLC (ACN/H_2_O, 40–60%) to obtain compounds **2** (13.4 mg), **5** (7.3 mg).

(2″*S*)-2″,3″-dihydrodelicaflavone (1): yellow amorphous powder; ${\left[\alpha \right]}_{D}^{25}$ –19.4 (*c* 0.08, MeOH); HPLC-UV (ACN-H_2_O) *λ*_max_: 279, 290, 340 nm; ^1^H NMR (500 MHz, DMSO-*d*_6_) data see Table 1, and ^13^C NMR (125 MHz, DMSO-*d*_6_) data see Table 2; HRESIMS *m*/*z* 541.1127 [M + H]^+^ (calcd. for C_30_H_21_O_10_, 541.1129).

6″-methyldelicaflavone (**2**): yellow amorphous powder; HPLC-UV (ACN-H_2_O) *λ*_max_: 265, 295, 340 nm; ^1^H NMR (500 MHz, DMSO-*d*_6_) data see Table 1, and ^13^C NMR (125 MHz, DMSO-*d*_6_) data see Table 2; HRESIMS *m*/*z* 553.1128 [M + H]^+^ (calcd. for C_31_H_21_O_10_, 553.1135).

7-O-methyldelicaflavone (**3**): yellow amorphous powder; HPLC-UV (ACN-H_2_O) *λ*_max_: 265, 293, 340 nm; ^1^H NMR (500 MHz, DMSO-*d*_6_) data see Table 1, and ^13^C NMR (125 MHz, DMSO-*d*_6_) data see Table 2; HRESIMS *m*/*z* 553.1119 [M + H]^+^ (calcd. for C_31_H_21_O_10_, 553.1129).

(2″*S*)-6″-methyl-2″,3″-dihydrodelicaflavone (4): yellow amorphous powder; ${\left[\alpha \right]}_{D}^{25}$ –14.2 (*c* 0.05, MeOH); HPLC-UV (ACN-H_2_O) *λ*_max_: 265, 295, 340 nm; ^1^H NMR (800 MHz, DMSO-*d*_6_) data see Table 1, and ^13^C NMR (200 MHz, DMSO-*d*_6_) data see Table 2; HRESIMS *m*/*z* 555.1299 [M + H]^+^ (calcd. for C_31_H_23_O_10_, 555.1286).

### 4.4. Cell Culture

Human lung carcinoma cell lines (A549), human lung adenocarinoma epithelial cell lines (H1299) and human normal embryonic lung cell lines (MRC-5) were purchased from the Chinese Academy of Sciences (Shanghai). H1299 and MRC-5 cells were maintained in RPMI1640 medium, and A549 cell lines were maintained in MEN medium. Both media were supplemented with 10% fetal bovine serum. All of the cell lines were grown at 37 °C in a 5% CO_2_ atmosphere. The cell lines mentioned above were checked for mycoplasma contamination, and no contamination was observed.

### 4.5. Cytotoxicity Assay

Cytotoxicity of test compounds was assessed by the MTT assay. A549, H1299 and MRC-5 cells at a final density (3000 cells/well) were placed in 96-well cell plates and treated with or without different concentrations of the test compounds for 24 h. At the end of the treatment, 3-(4,5-dimethyl-2-thiazolyl)-2,5-diphenyl-2-H-tetrazolium bromide (MTT, 20 mL, 5 mg/mL) was added into each well, and the cells were further incubated for 4 h. The resulting formazan crystals were dissolved in 150 mL of DMSO and the absorbance was read spectrophotometrically at 570 nm using an enzyme-linked immunosorbent assay plate reader. Experiments were conducted in triplicate. Inhibition rate (%) = [(Acontrol − Atreated)/Acontrol] × 100%. IC_50_ value represents the half of maximal inhibitory concentration.

### 4.6. Morphological Observations

A549 cells were plated in 6 well culture plates with a cell density of 1 × 10^5^ cells/mL and allowed to adhere for 12 h. The cells were incubated with vehicle control (i.e., 1% DMSO in buffer) or compounds **1**–**3** at 3 μM for 24 h treatment, respectively. Cell morphological features were photographed with a phase-contrast microscope. The quantitative analysis of live cells was determined by Image J software.

### 4.7. Apoptosis Assay

Cells were incubated in six-well plates (1 × 10^5^/well) and treated with vehicle control (i.e., 1% DMSO in buffer) or compounds **1**–**3** at 3 μM for 24 h, respectively. The collected cells were harvested, washed with PBS, and then stained with FITC-Annexin-V and PI. Apoptosis was determined with flow cytometry.

### 4.8. Cell Cycle Analysis

A549 cells were treated with vehicle control (i.e., 1% DMSO in buffer) or compound **3** at indicated concentration for 24 h, respectively. The cells were harvested, washed, then fixed with 70% ethanol at −20 °C for 8 h, and incubated with PI/RNase staining buffer (BD Pharmingen) for 15 min at room temperature (25 °C). The DNA content in the different groups of cells was assessed with flow cytometry and analyzed by the software MODFIT.

### 4.9. Western Blotting Assay

A549 cells (1 × 10^5^/well) were cultured in six-well plates overnight and treated in triplicate with vehicle DMSO (0.1%, *v*/*v*) alone, or with indicated compound at 3 μM for 24 h. The cells were harvested and lysed with cell lysis buffer containing protease and phosphatase inhibitor cocktails (Beyotime, Shanghai, China). The concentrations of protein were determined using a BCA protein assay kit (Beyotime, Shanghai, China) and equalized before loading. Samples were denatured and subjected to SDS-PAGE gels followed by transfer to PVDF membrane and probed with specific antibodies against cleaved caspase-3, caspase-3, survivin, XIAP, β-actin, and GAPDH (Abcam, Shanghai, China), and then incubated with the secondary antibodies diluted in TBST. Finally, the protein bands were detected with ECL Western Blotting Kit according to the manufacturer’s specifications. Eventually, films were obtained and the light density of the target bar was analyzed by Image J software.

### 4.10. Statistical Analysis

All statistical analyses were performed using the GraphPad Prism software version 5.0 (GraphPad Software Inc., La Jolla, CA, USA). Statistically significant differences were analyzed using the non-parametric tests (Mann–Whitney), and *p*-values of less than 0.05 were considered to indicate statistical significance. Data are expressed as means from three independent experiments.

## Figures and Tables

**Table 1:** ^1^H NMR spectral data for compounds **1**–**4** in DMSO-*d*_6_ (*δ* in ppm, and *J* in Hz).

Position	1 ^a^	2 ^a^	3 ^a^	4 ^b^
6	6.24, d (2.0)	6.05, d (2.0)	6.26, d (2.0)	6.24, d (1.6)
8	6.54, d (2.0)	6.32, d (2.0)	6.55, d (2.0)	6.54, d (1.6)
7-OCH_3_			3.87, s	
2′/6′	7.87, d (8.5)	7.78, d (9.0)	7.84, d (9.0)	7.87, d (8.8)
3′/5′	6.89, d (8.5)	6.84, d (9.0)	6.89, d (9.0)	6.89, d (8.8)
2″	5.50, dd (13.0, 3.0)			5.46, dd (12.8, 2.4)
3″	3.26, dd (17.0, 3.0)	6.77, s	6.95, s	3.24, dd (16.8, 2.4)
	2.71, dd (17.0, 3.0)			2.72, dd, (16.8, 2.4)
6″	5.89, d (2.0)		6.40, d (2.0)	
8″	5.90, d (2.0)	6.43, s	6.78, d (2.0)	5.99, s
6″-CH_3_		1.97, s		1.88, s
2′′′/6′′′	7.45, d (8.5)	7.95, d (8.5)	8.04, d (9.0)	7.44, d (8.8)
3′′′/5′′′	7.10, d (8.5)	7.19, d (8.5)	7.27, d (9.0)	7.10, d (8.8)
5-OH	12.30, s	12.21, br s	12.24, s	12.31, br s
7-OH	10.96, br s			10.89, br s
4′-OH	10.37, br s		10.38, br s	10.39, br s
5″-OH	12.14, s	13.07, br s	12.88, s	12.40, s
7″-OH	10.82, br s		11.04, br s	10.89, br s

^a^ Measured at 500 MHz; ^b^ Measured at 800 MHz.

**Table 2:** ^13^C NMR spectral data for compounds **1**–**4** in DMSO-*d*_6_.

Position	1 ^a^	2 ^a^	3 ^a^	4 ^b^
2	160.7	161.5	160.8	161.1
3	132.1	131.8	131.8	132.4
4	176.4	175.6	176.1	176.8
5	161.3	161.5	161.3	161.6
6	99.0	100.7	99.1	99.3
7	164.6	163.2	164.7	165.0
8	94.2	95.3	93.0	94.6
9	156.9	156.6	156.9	157.2
10	104.3	104.4	104.3	104.6
7-OCH_3_			56.2	
1′	120.0	120.0	119.9	120.3
2′/6′	130.3	130.4	130.3	130.6
3′/5′	115.9	116.3	115.9	116.2
4′	157.1	155.8	157.2	157.5
2″	78.2	162.6	163.5	78.5
3″	42.1	104.4	104.5	42.5
4″	196.2	181.7	182.2	196.7
5″	163.6	158.8	161.3	161.2
6″	96.0	107.8	98.2	103.8
7″	166.8	165.4	165.4	165.1
8″	95.1	93.8	94.4	94.7
9″	163.0	157.6	157.5	160.7
10″	101.9	103.3	104.9	101.9
6″-CH_3_		8.0		7.4
1′′′	132.7	125.5	125.0	133.2
2′′′/6′′′	128.6	128.7	128.8	128.9
3′′′/5′′′	115.1	116.1	115.9	115.4
4′′′	157.1	160.0	159.8	157.2

^a^ Measured at 125 MHz; ^b^ Measured at 200 MHz.

**Table 3:** Antiproliferative activity of compounds **1**–**5**.

Compounds	IC_50_ (μM) ^a^		
	A549	H1299	MRC-5
**1**	3.1 ^c,d^	7.3 ^c,d^	>100
**2**	2.8 ^c,d^	7.3 ^c,d^	>100
**3**	2.3 ^c,d^	4.0 ^c,d^	>100
**4**	3.1 ^c,d^	6.8 ^c,d^	58.2
**5**	7.9 ^c,d^	8.4 ^c,d^	36.8
DDP ^b^	13.3	24.5	49.0

^a^ IC_50_ values represent the mean of independent triplicate experiments (*n* = 3), and the statistically significant differences were analyzed using Mann-Whitney; ^b^ DDP: *Cis*-platin used as a positive control; ^c^ *p* < 0.001 vs the DDP group; ^d^ *p* < 0.001 vs the MRC-5 group.

## Data Availability

All data are available in this publication and in the Supplementary Materials.

## Supplementary Materials

The following are available online, Figure S1: HRESIMS spectrum of **1**, Figure S2: ^1^H NMR spectrum (500 MHz) of **1** in DMSO-*d_6_*, Figure S3: ^13^C NMR spectrum (125 MHz) of **1** in DMSO-*d*_6_, Figure S4: DEPT 135 spectrum (125 MHz) of **1** in DMSO-*d*_6_, Figure S5: ^1^H-^1^H COSY spectrum of **1** in DMSO-*d*_6_, Figure S6: HSQC spectrum of **1** in DMSO-*d*_6_, Figure S7: HMBC spectrum of **1** in DMSO-*d*_6_, Figure S8: HRESIMS spectrum of **2**, Figure S9: ^1^H NMR spectrum (500 MHz) of **2** in DMSO-*d_6_*, Figure S10: ^13^C NMR spectrum (125 MHz) of **2** in DMSO-*d*_6_, Figure S11: DEPT 135 spectrum (125 MHz) of **2** in DMSO-*d*_6_, Figure S12: ^1^H-^1^H COSY spectrum of **2** in DMSO-*d*_6_, Figure S13: HSQC spectrum of **2** in DMSO-*d*_6_, Figure S14: HMBC spectrum of **2** in DMSO-*d*_6_, Figure S15: HRESIMS spectrum of **3**, Figure S16: ^1^H NMR spectrum (500 MHz) of **3** in DMSO-*d_6_*, Figure S17: ^13^C NMR spectrum (125 MHz) of **3** in DMSO-*d*_6_, Figure S18: DEPT 135 spectrum (125 MHz) of **3** in DMSO-*d*_6_, Figure S19: ^1^H-^1^H COSY spectrum of **3** in DMSO-*d*_6_, Figure S20: HSQC spectrum of **3** in DMSO-*d*_6_, Figure S21: HMBC spectrum of **3** in DMSO-*d*_6_, Figure S22: HRESIMS spectrum of **4**, Figure S23: ^1^H NMR spectrum (800 MHz) of **4** in DMSO-*d_6_*, Figure S24: ^13^C NMR spectrum (200 MHz) of **4** in DMSO-*d*_6_, Figure S25: DEPT 135 spectrum (125 MHz) of **4** in DMSO-*d*_6_, Figure S26: ^1^H-^1^H COSY spectrum of **4** in DMSO-*d*_6_, Figure S27: HSQC spectrum of **4** in DMSO-*d*_6_, Figure S28: HMBC spectrum of **4** in DMSO-*d*_6_, Figure S29: Effects of compounds **1–3** on expression of caspase-3/cleaved caspase-3.

## Sample Availability

Samples of the compounds are available from the authors.
