# Supplementary material for: Biflavonoids from Selaginella doederleinii as Potential Antitumor Agents for Intervention of Non-Small Cell Lung Cancer
Source: Molecules. 2021 Sep 5;26(17):5401. doi: 10.3390/molecules26175401 (PMC8434134; doi:10.3390/molecules26175401)
Supplement: Supplementary file 1 [file molecules-26-05401-s001.zip › molecules-1348945-supplementary.pdf]

Supplementary Information

Biflavonoids from *Selaginella doederleinii* as potential antitumor agents for intervention of non-small-cell lung cancer

Fenghua Kang, [1,2,3] Sha Zhang, [1,2,3] Dekun Chen, [1,2,3] Jianbing Tan, [1,2,3] Min Kuang, [1,2,3] Jinlin Zhang, [1,2,3] Guangyuan Zeng, [1,2,3] Kangping Xu, [1,2,3] Zhenxing Zou [1,2,3*] and Guishan Tan [1,2,3*]

[1] *Xiangya School of Pharmaceutical Sciences, Central South University, Changsha 410013, PR China.*

[2] *Xiangya Hospital of Central South University, Changsha 410008, PR China.*

[3] *Hunan Key laboratory of Diagnostic and Therapeutic Drug Research for Chronic Diseases, Central South University, Changsha, 410013, Hunan, China.*

Content .... page

(2"*S*)-2",3"-dihydrodelicaflavone (1)

Figure S1. HRESIMS spectrum of **1** 1

Figure S2. ^{1}H NMR spectrum (500 MHz) of **1** in DMSO-d_6 1

Figure S3. ^{13}C NMR spectrum (125 MHz) of **1** in DMSO-d_6 2

Figure S4. DEPT 135 spectrum (125 MHz) of **1** in DMSO-d_6 2

Figure S5. ^{1}H-^{1}H COSY spectrum of **1** in DMSO-d_6 3

Figure S6. HSQC spectrum of **1** in DMSO-d_6 3

Figure S7. HMBC spectrum of **1** in DMSO-d_6 4

6"-methyldelicaflavone (2)

Figure S8. HRESIMS spectrum of **2** 4

Figure S9. ^{1}H NMR spectrum (500 MHz) of **2** in DMSO-d_6 5

Figure S10. ^{13}C NMR spectrum (125 MHz) of **2** in DMSO-d_6 5

Figure S11. DEPT 135 spectrum (125 MHz) of **2** in DMSO-d_6 6

Figure S12. ^{1}H-^{1}H COSY spectrum of **2** in DMSO-d_6 6

Figure S13. HSQC spectrum of **2** in DMSO-d_6 7

Figure S14. HMBC spectrum of **2** in DMSO-d_6 7

7-*O*-methyldelicaflavone (3)

Figure S15. HRESIMS spectrum of **3** 8

Figure S16. ^{1}H NMR spectrum (500 MHz) of **3** in DMSO-d_6 8

Figure S17. ^{13}C NMR spectrum (125 MHz) of **3** in DMSO-d_6 9

Figure S18. DEPT 135 spectrum (125 MHz) of **3** in DMSO-d_6 9

Figure S19. ^{1}H-^{1}H COSY spectrum of **3** in DMSO-d_6 10

Figure S20. HSQC spectrum of **3** in DMSO-d_6 10

Figure S21. HMBC spectrum of **3** in DMSO-d_6 11

(2"*S*)-6"-methyl-2",3"-dihydrodelicaflavone (4)

Figure S22. HRESIMS spectrum of **4** 11

Figure S23. ^{1}H NMR spectrum (800 MHz) of **4** in DMSO-d_6 12

Figure S24. ^{13}C NMR spectrum (200 MHz) of **4** in DMSO-d_6 12

Figure S25. DEPT 135 spectrum (125 MHz) of **4** in DMSO-d_6........................13

Figure S26. ^{1}H-^{1}H COSY spectrum of **4** in DMSO-d_6......................................13

Figure S27. HSQC spectrum of **4** in DMSO-d_6...14

Figure S28. HMBC spectrum of **4** in DMSO-d_6..14

Figure S29. Effects of compounds **1-3** on expression of caspase-3/cleaved caspase-3. 15

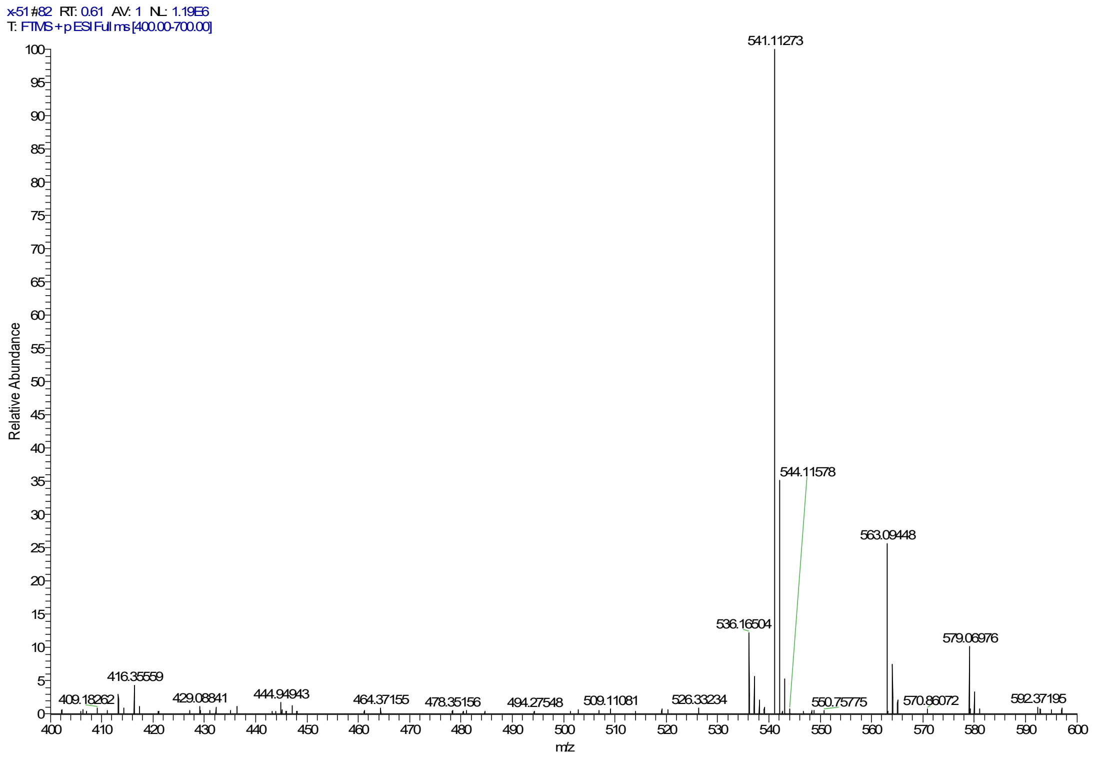

m/z Theo.	Mass	Delta (mmu)	RDB equiv.	Composition
541.11273	541.11292	-0.19	20.5	$C_{30}H_{21}O_{10}$

Figure S1. HRESIMS spectrum of **1**

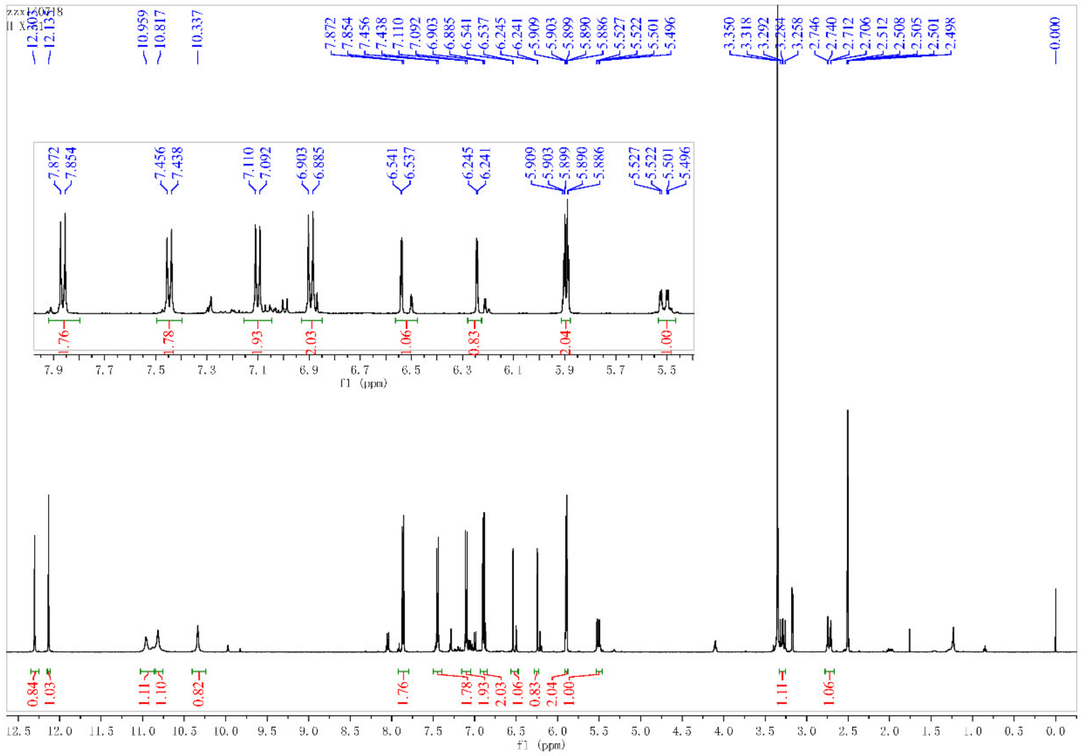

Figure S2. ^{1}H NMR spectrum (500 MHz) of **1** in DMSO-d_6

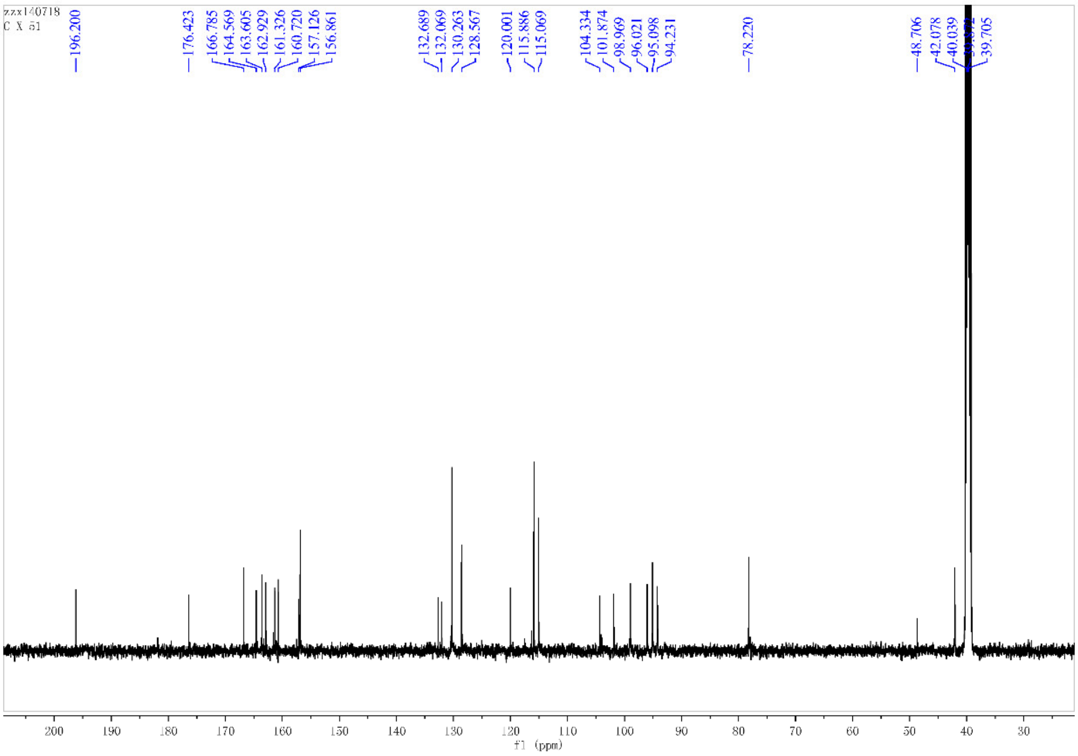

Figure S3. ^{13}C NMR spectrum (125 MHz) of **1** in DMSO-d_6

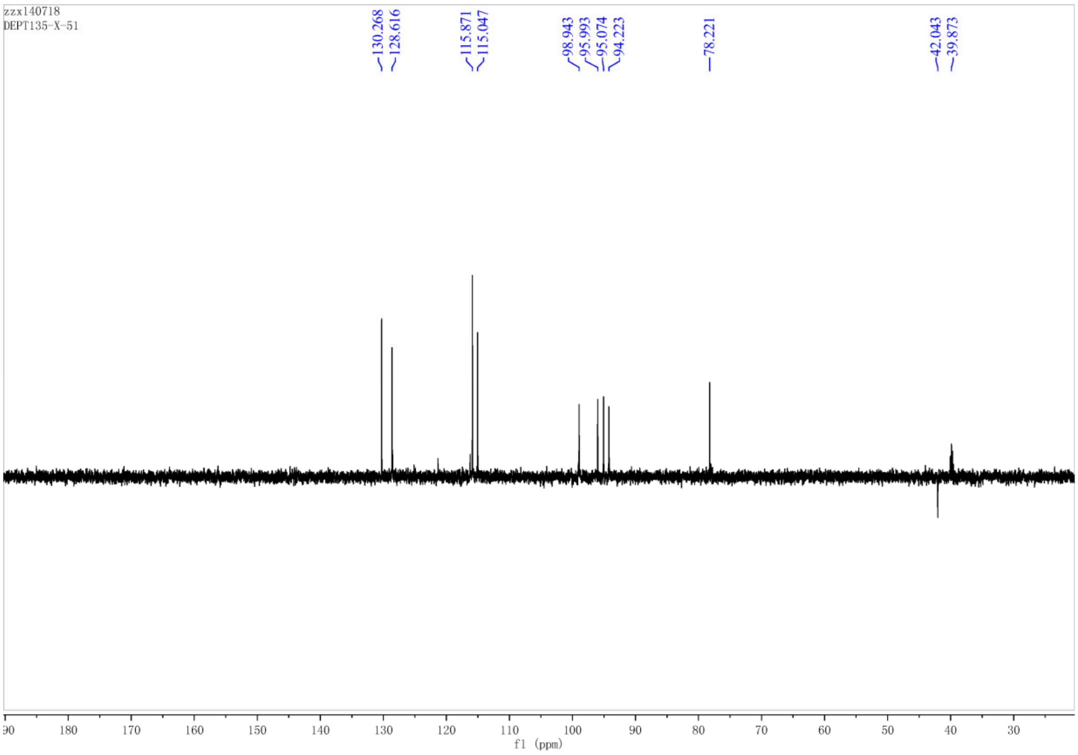

Figure S4. DEPT 135 spectrum (125 MHz) of **1** in DMSO-d_6

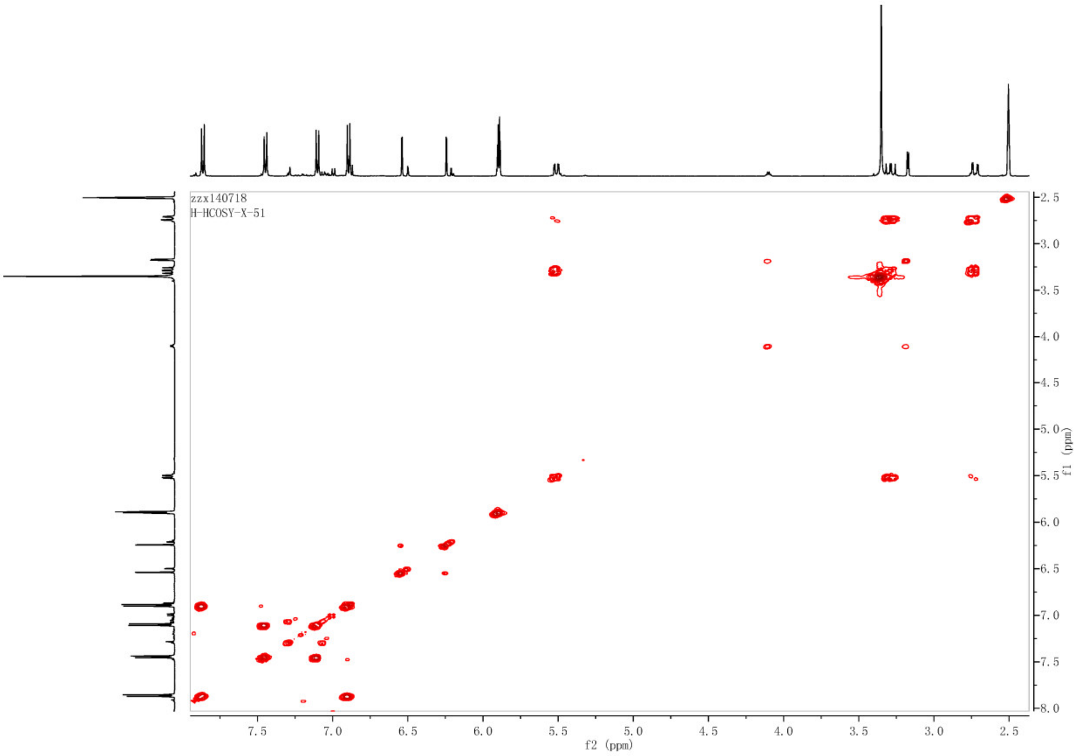

Figure S5. ^{1}H-^{1}H COSY spectrum of **1** in DMSO-d_6

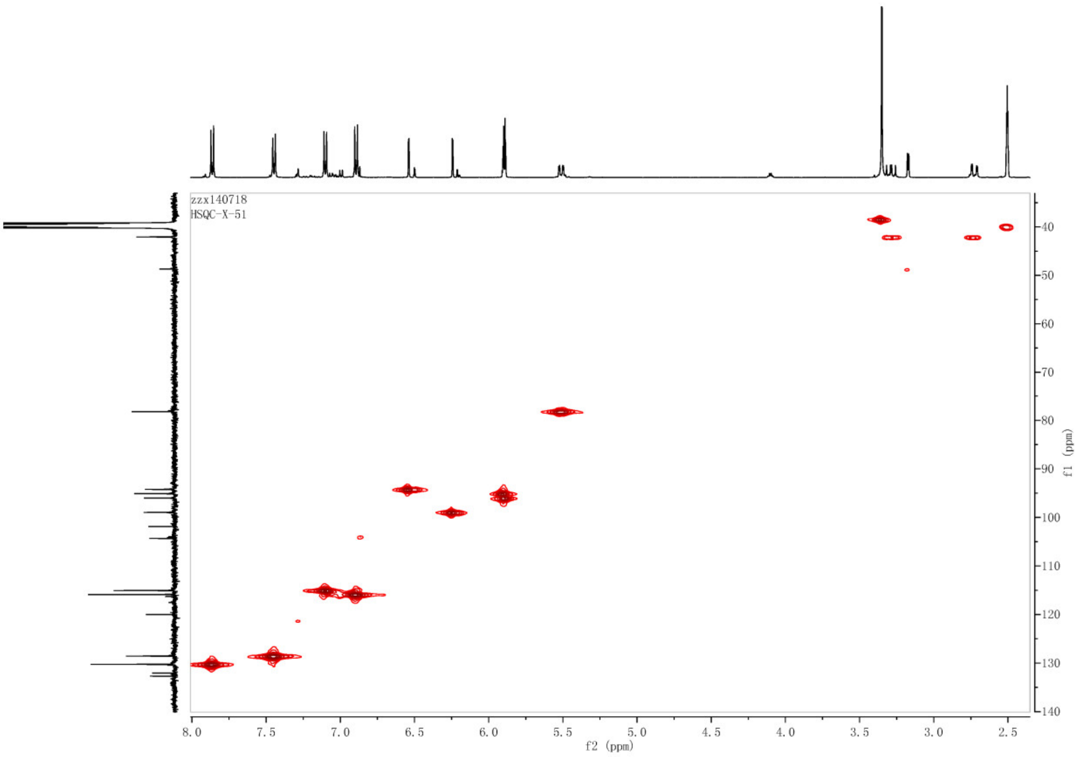

Figure S6. HSQC spectrum of **1** in DMSO-d_6

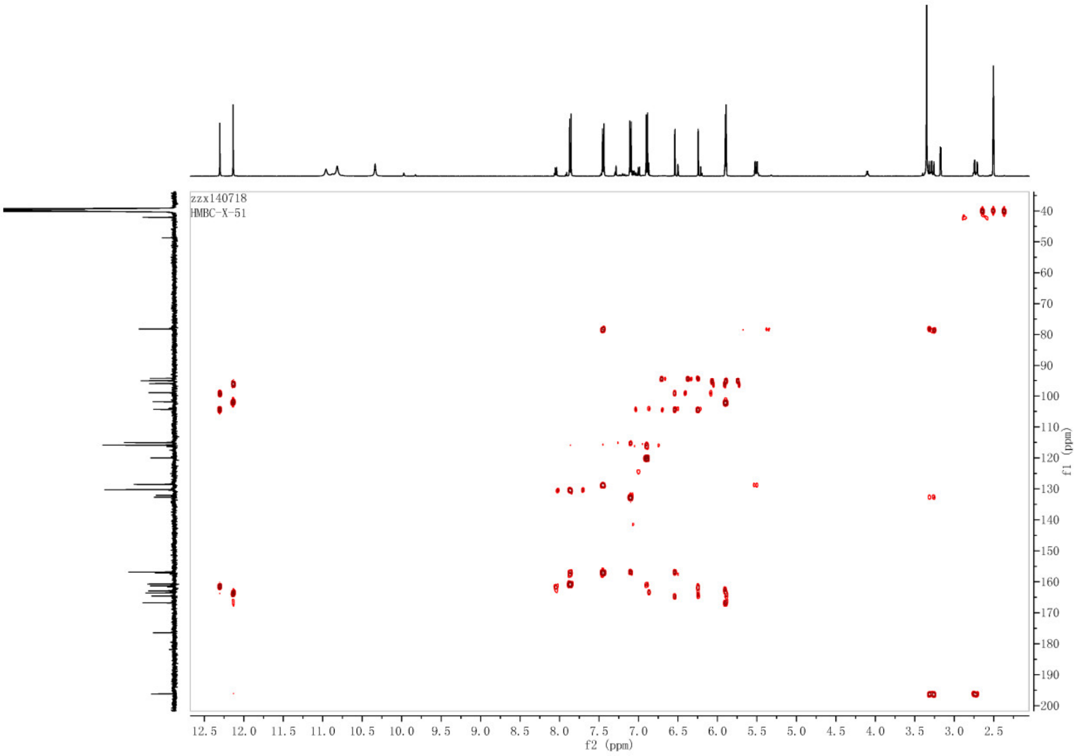

Figure S7. HMBC spectrum of **1** in DMSO-d_6

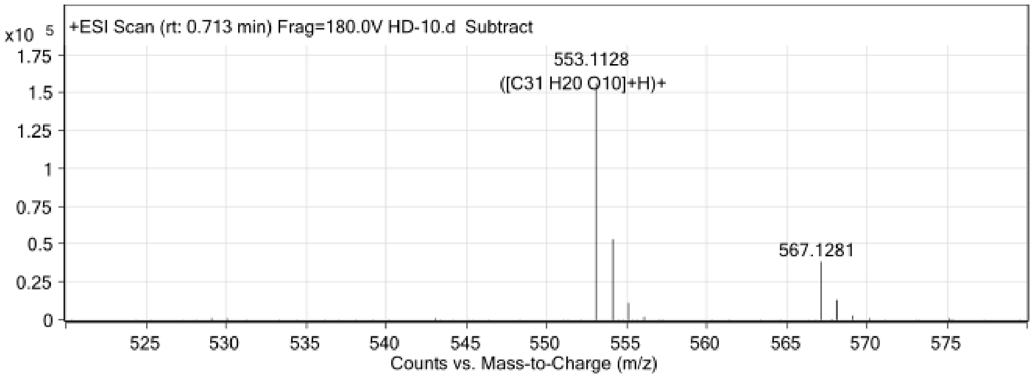

Figure S8. HRESIMS spectrum of **2**

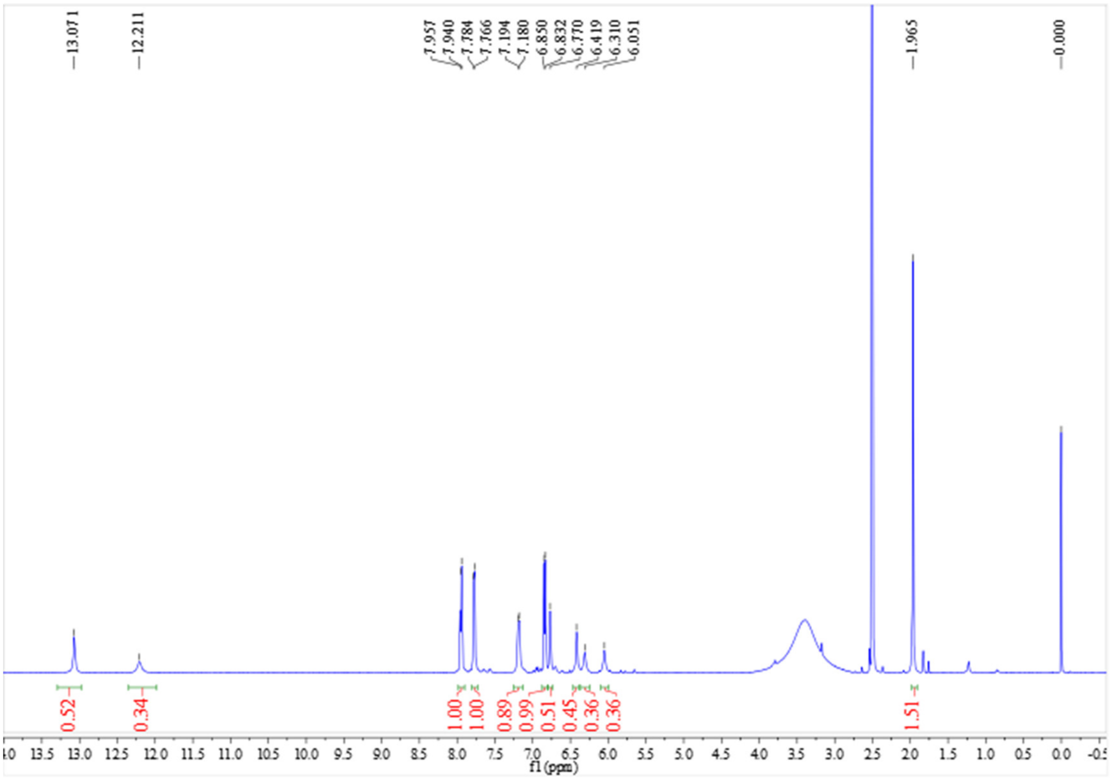

Figure S9. ^{1}H NMR spectrum (500 MHz) of **2** in DMSO-d_6

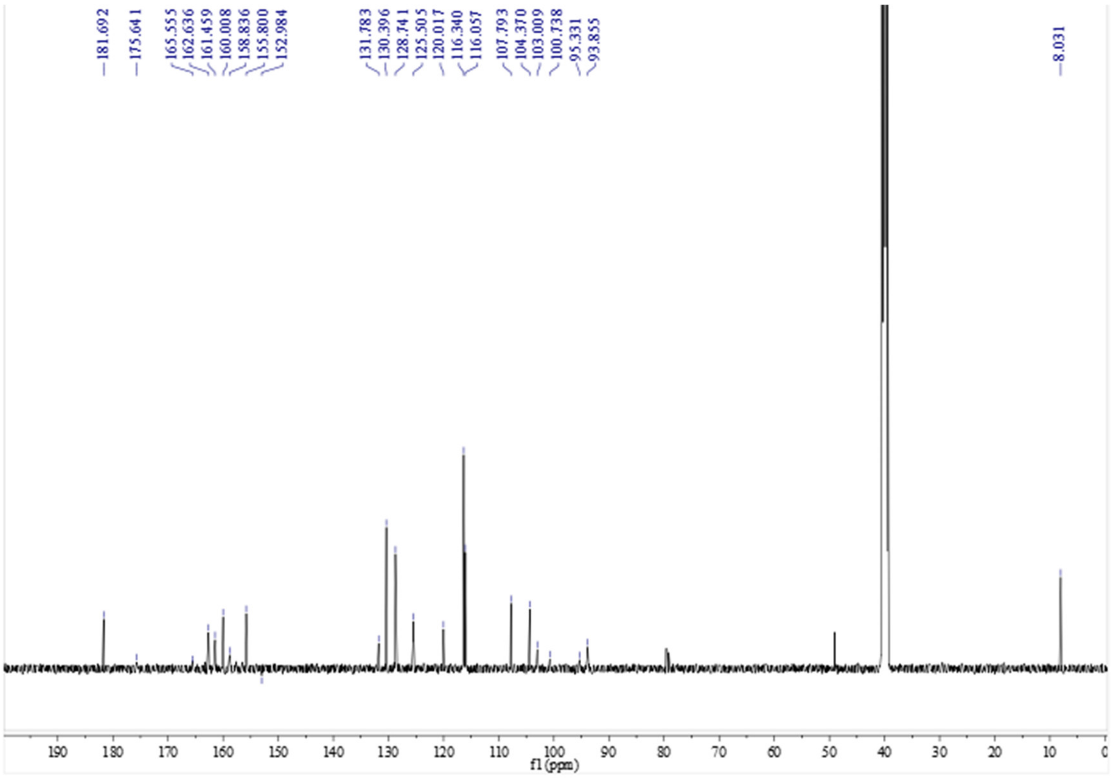

Figure S10. ^{13}C NMR spectrum (125 MHz) of **2** in DMSO-d_6

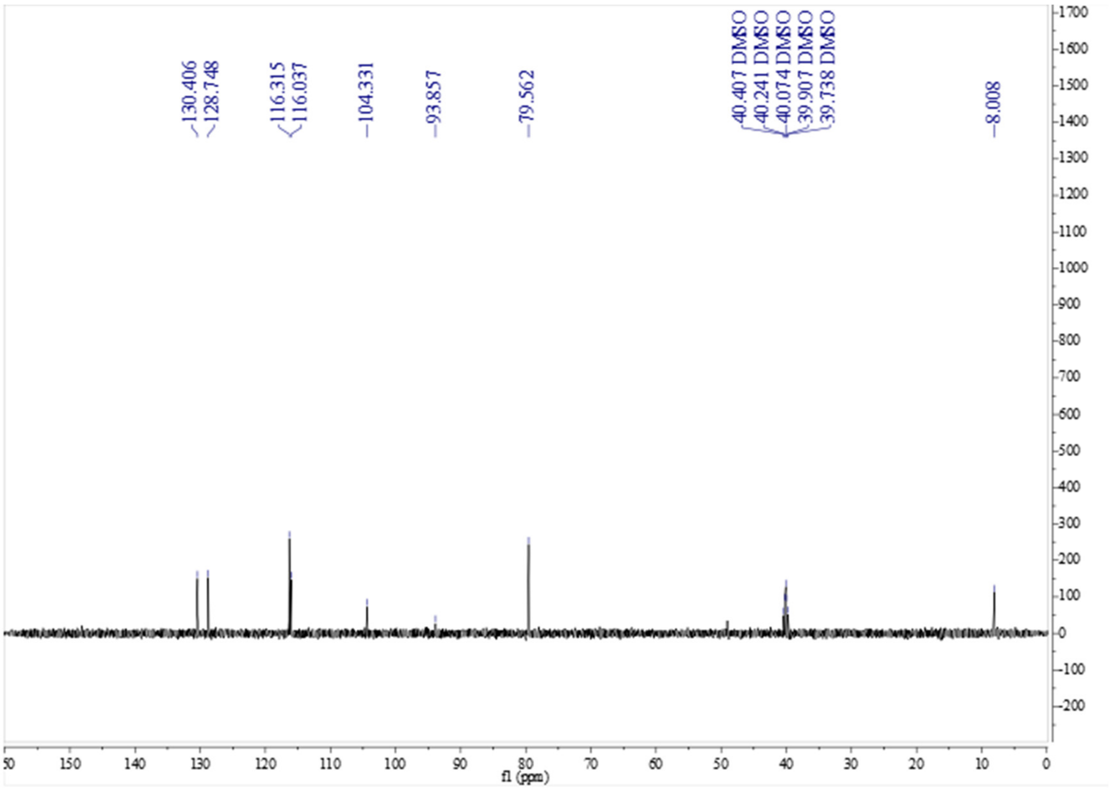

Figure S11. DEPT 135 spectrum (125 MHz) of **2** in DMSO-d_6

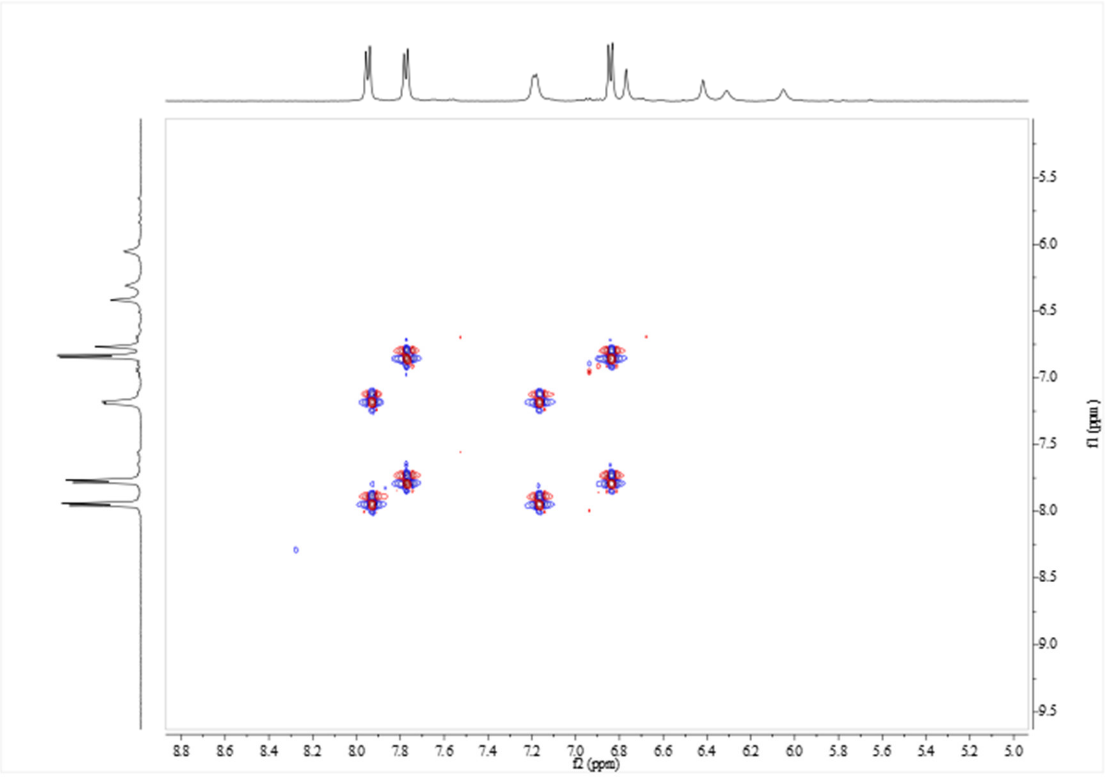

Figure S12. ^{1}H-^{1}H COSY spectrum of **2** in DMSO-d_6

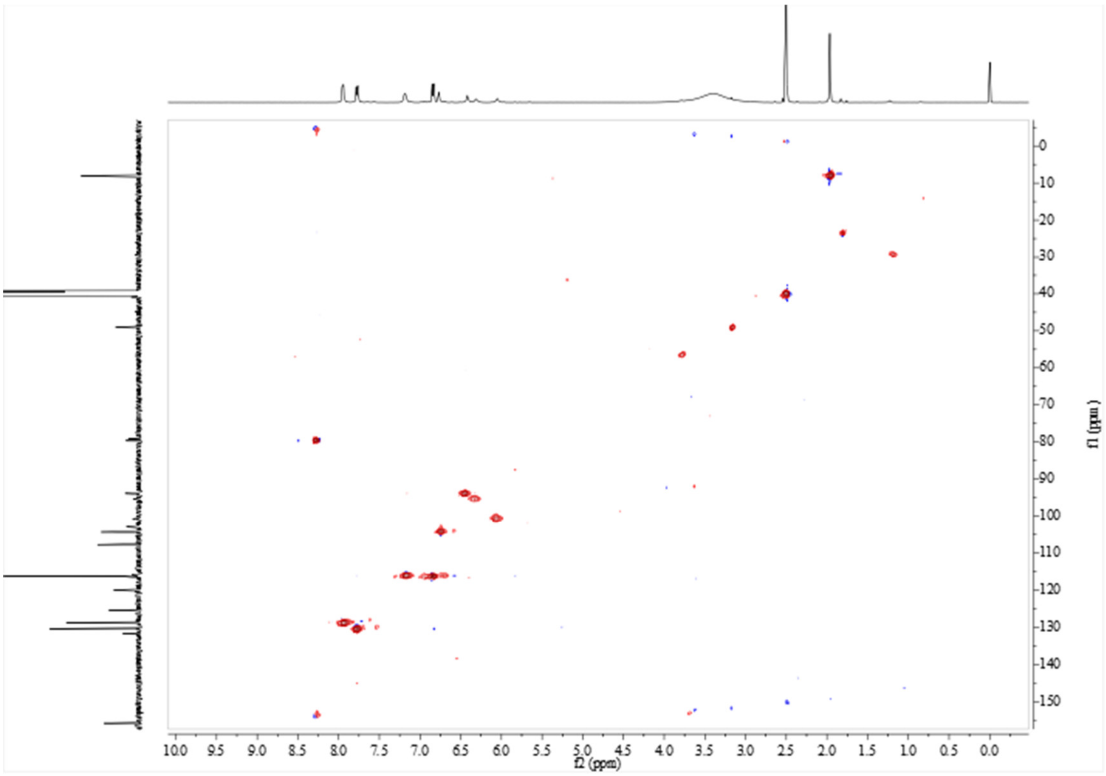

Figure S13. HSQC spectrum of **2** in DMSO-*d*6

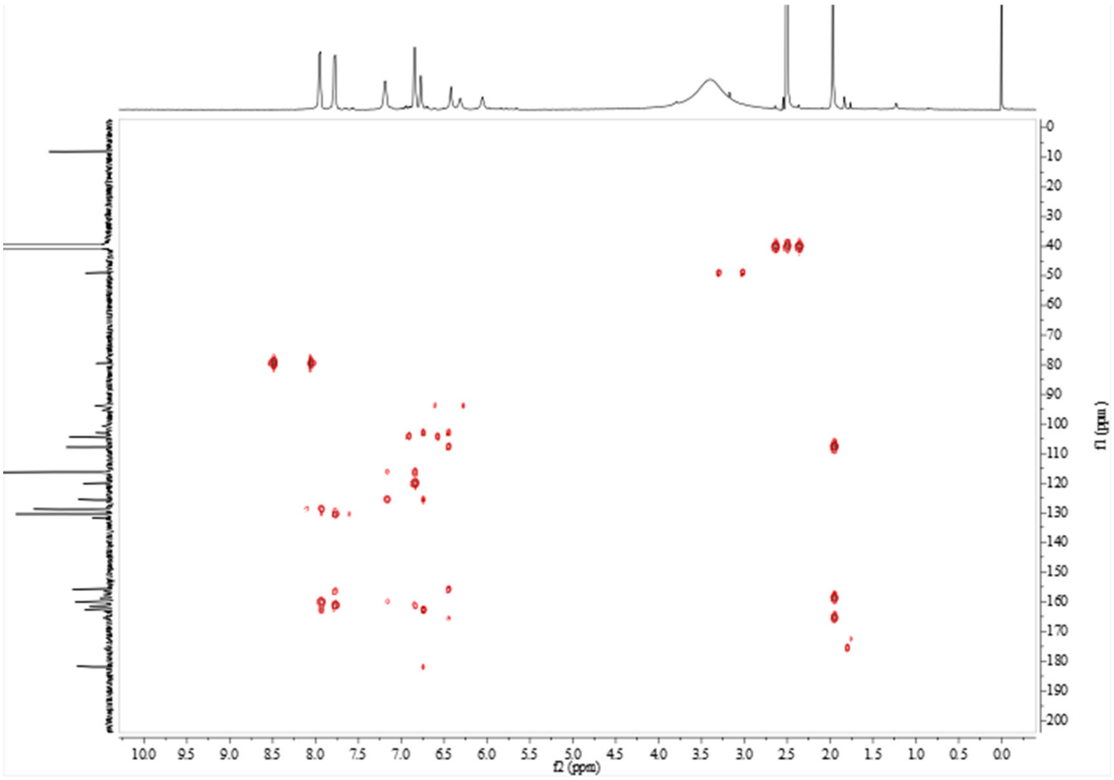

Figure S14. HMBC spectrum of **2** in DMSO-*d*6

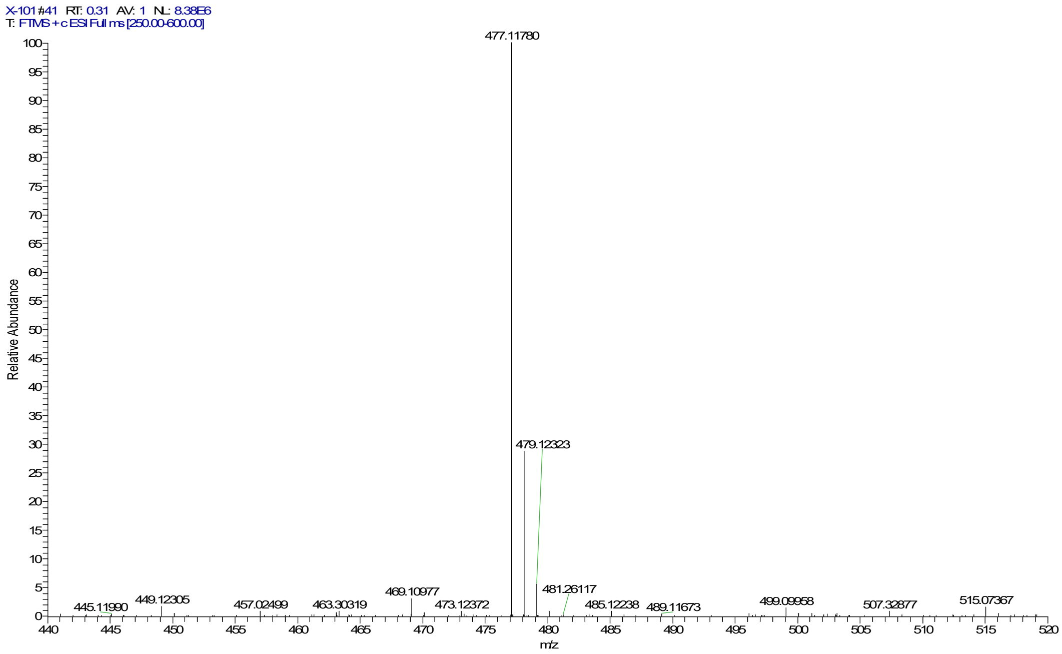

m/z Theo.	Mass	Delta (mmu)	RDB equiv.	Composition
477.11780	477.11801	-0.21	16.5	$C_{26}H_{21}O_{9}$

Figure S15. HRESIMS spectrum of **3**

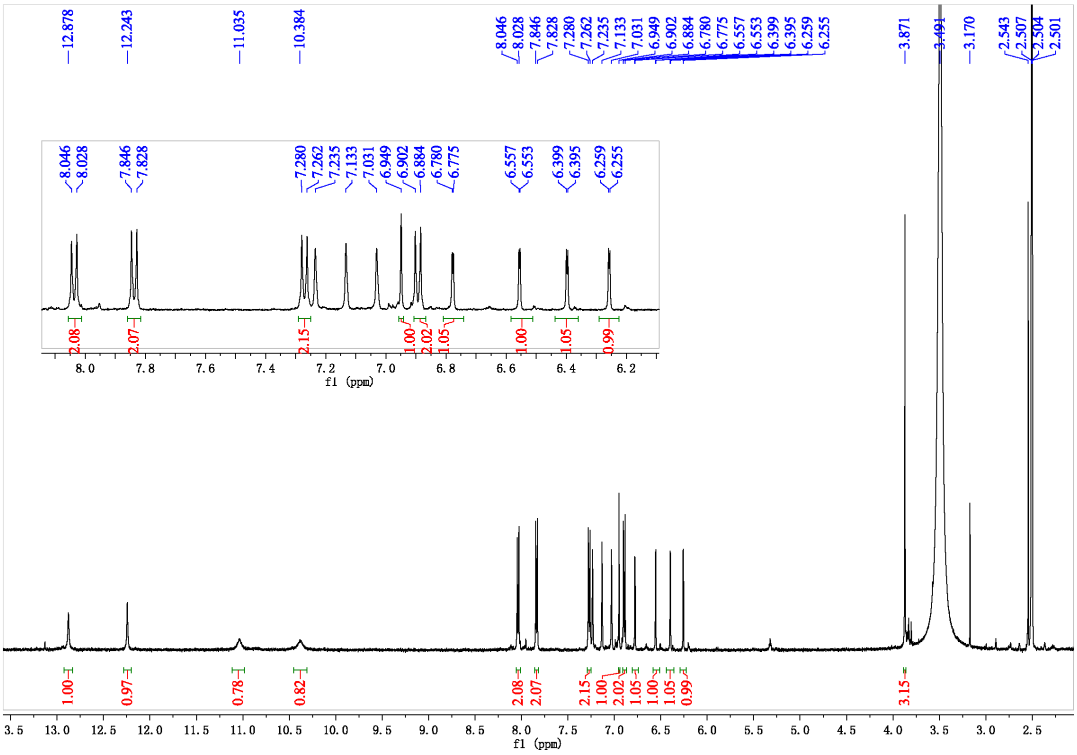

Figure S16. ^{1}H NMR spectrum (500 MHz) of **3** in DMSO-d_6

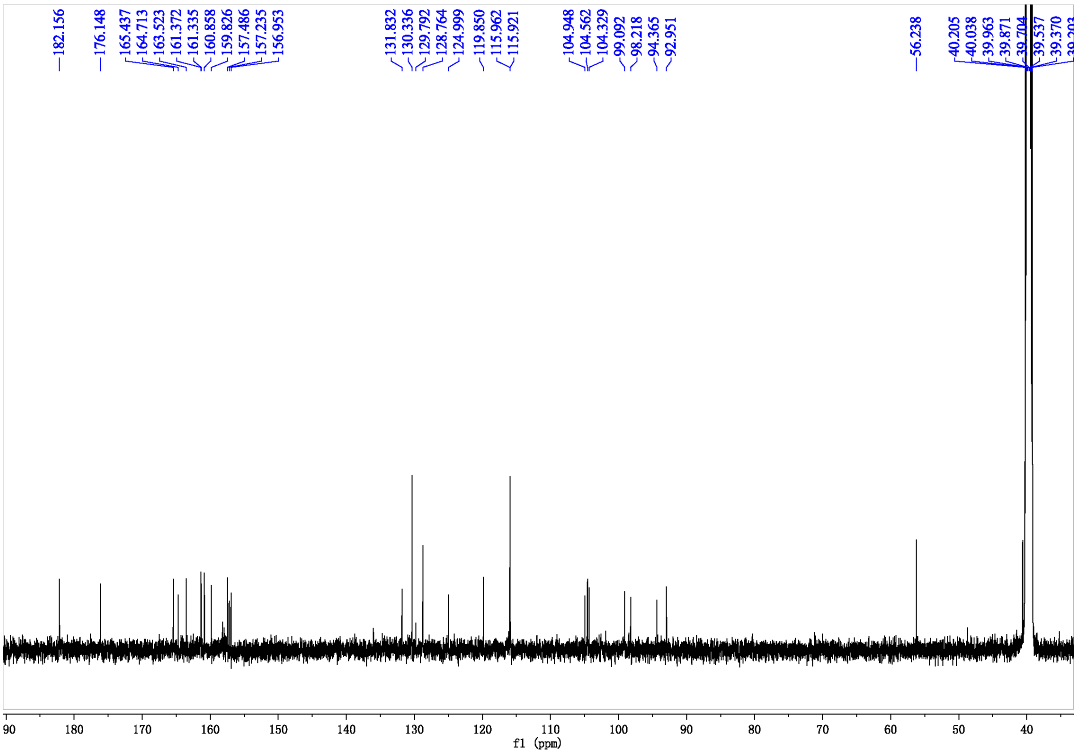

Figure S17. ^{13}C NMR spectrum (125 MHz) of **3** in DMSO-*d*6

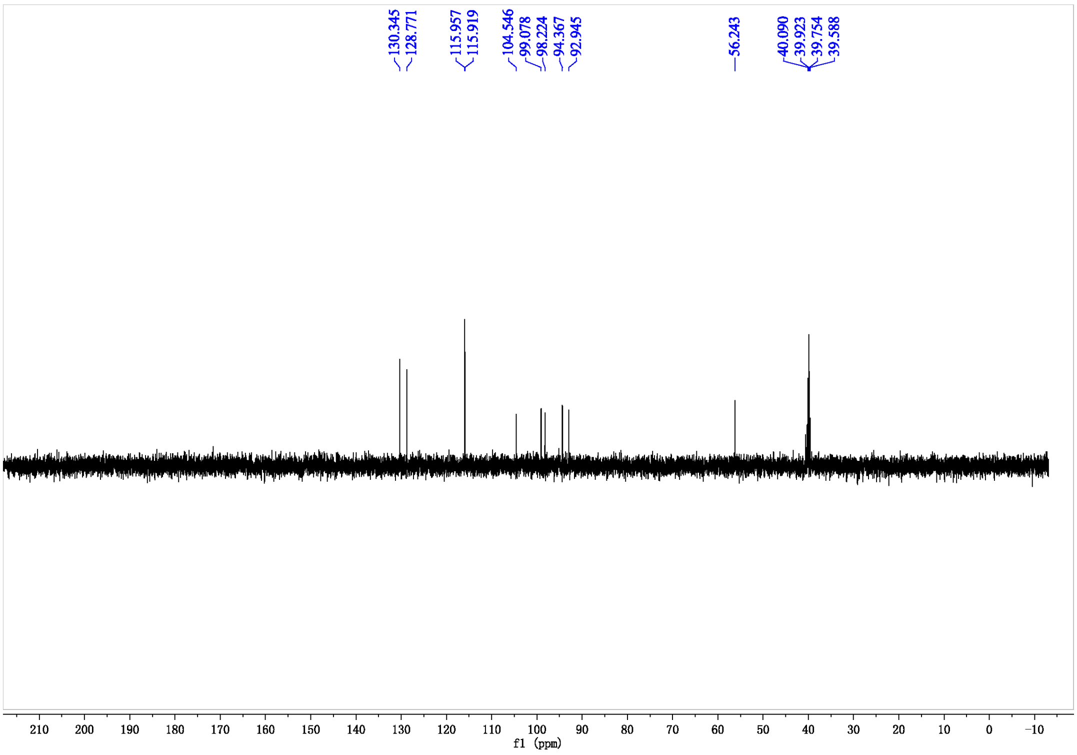

Figure S18. DEPT 135 spectrum (125 MHz) of **3** in DMSO-*d*6

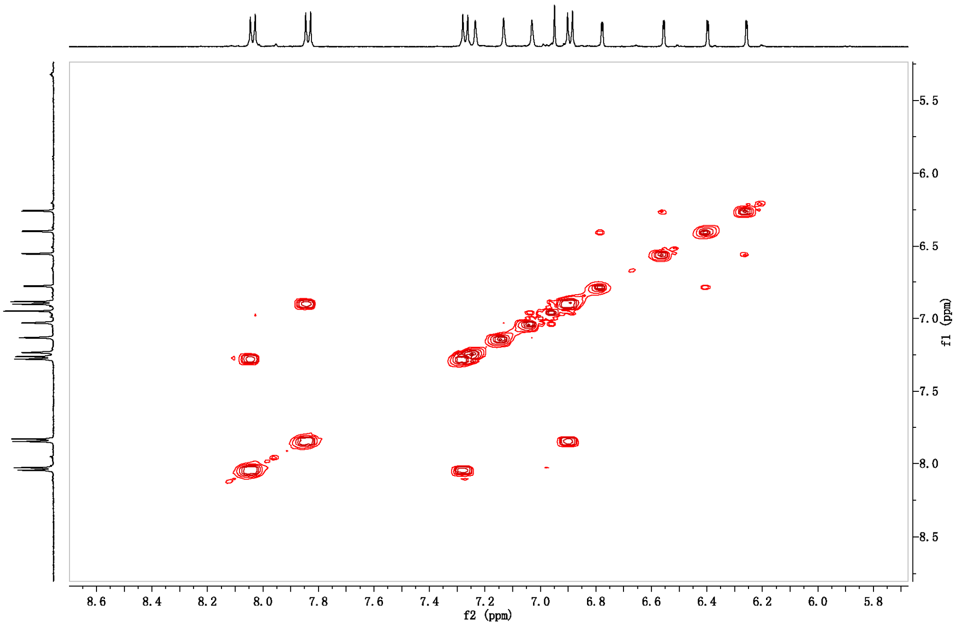

Figure S19. ^{1}H-^{1}H COSY spectrum of **3** in DMSO-*d*6

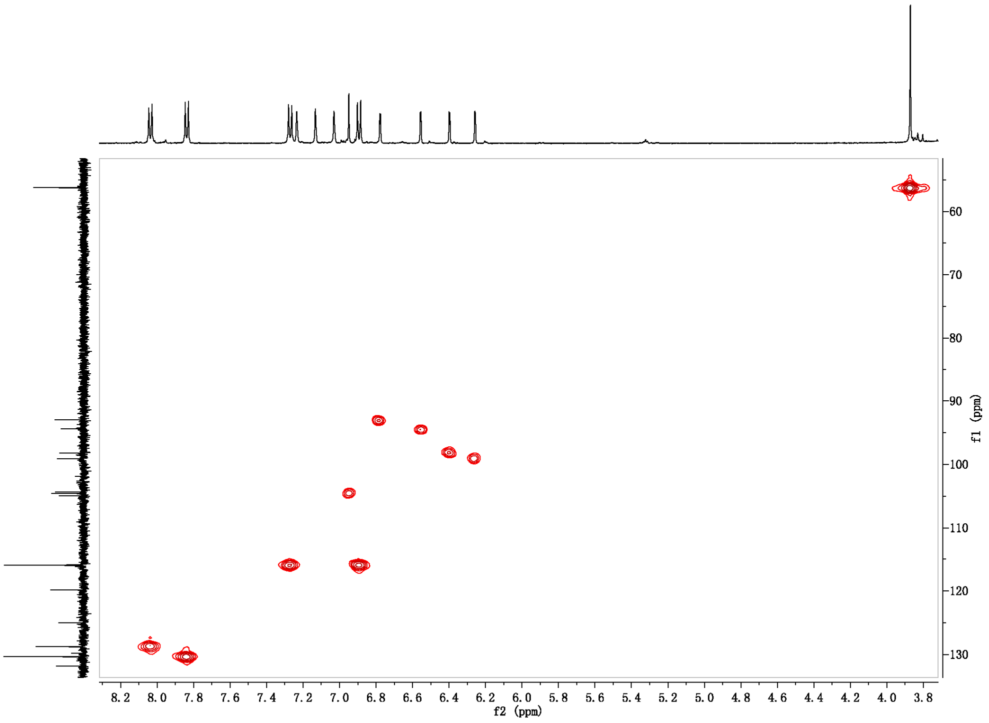

Figure S20. HSQC spectrum of **3** in DMSO-*d*6

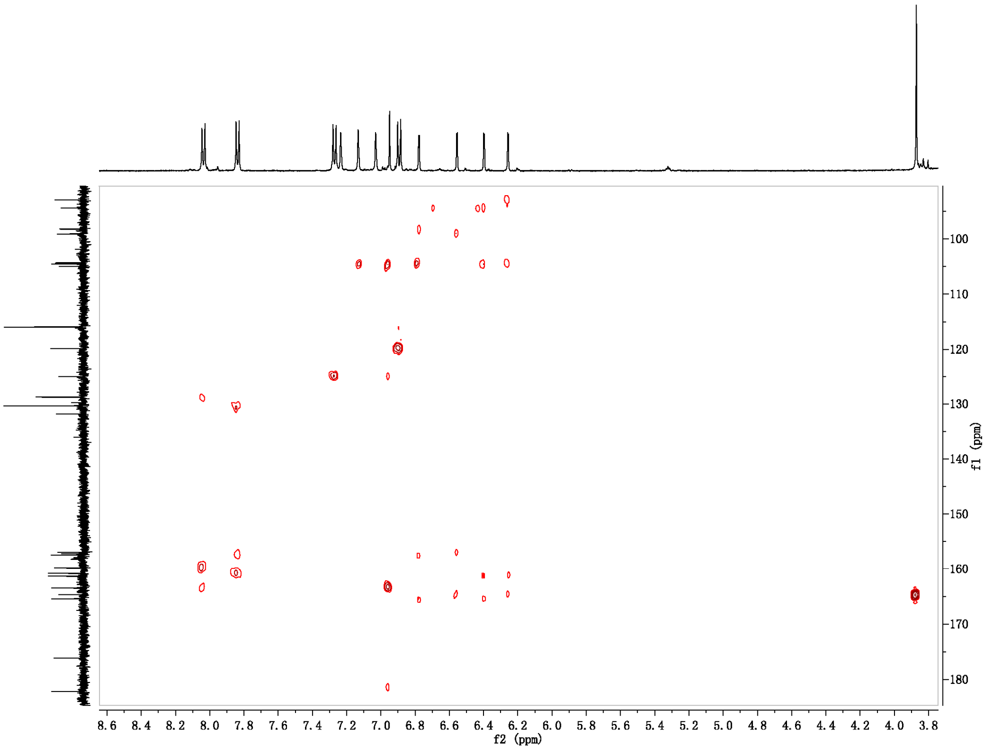

Figure S21. HMBC spectrum of **3** in DMSO-d_6

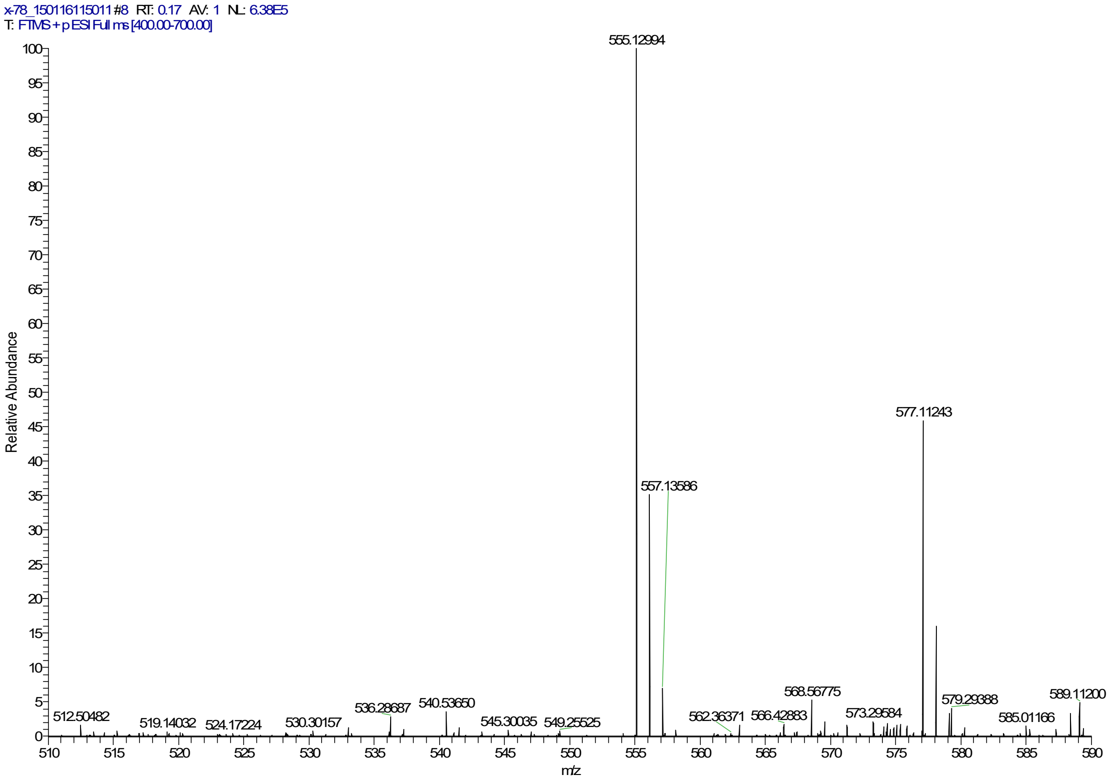

m/z Theo.	Mass	Delta (mmu)	RDB equiv.	Composition
555.12994	555.12857	1.37	20.5	$C_{31}H_{23}O_{10}$

Figure S22. HRESIMS spectrum of **4**

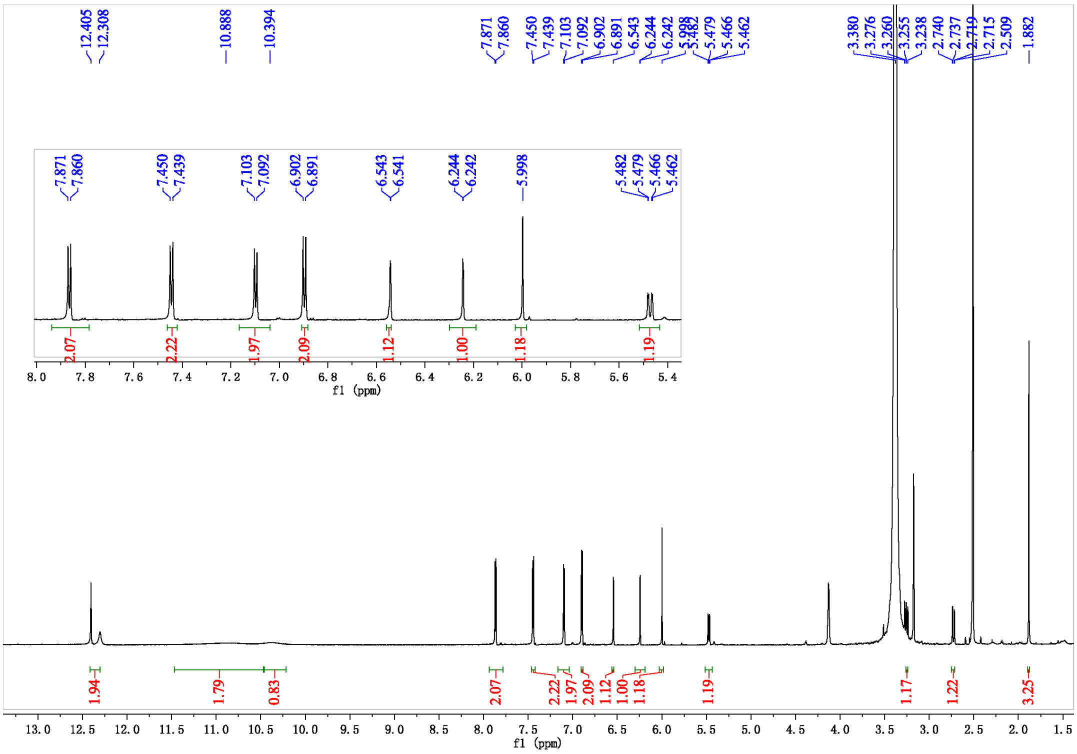

Figure S23. ^{1}H NMR spectrum (800 MHz) of **4** in DMSO-d_6

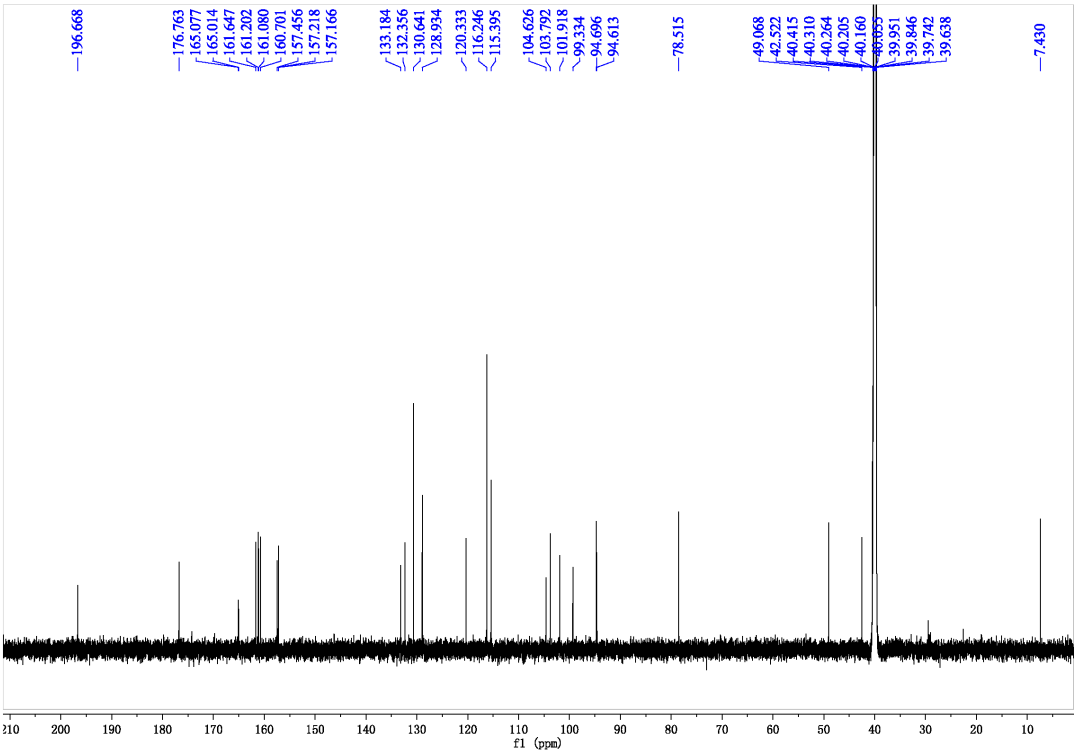

Figure S24. ^{13}C NMR spectrum (200 MHz) of **4** in DMSO-d_6

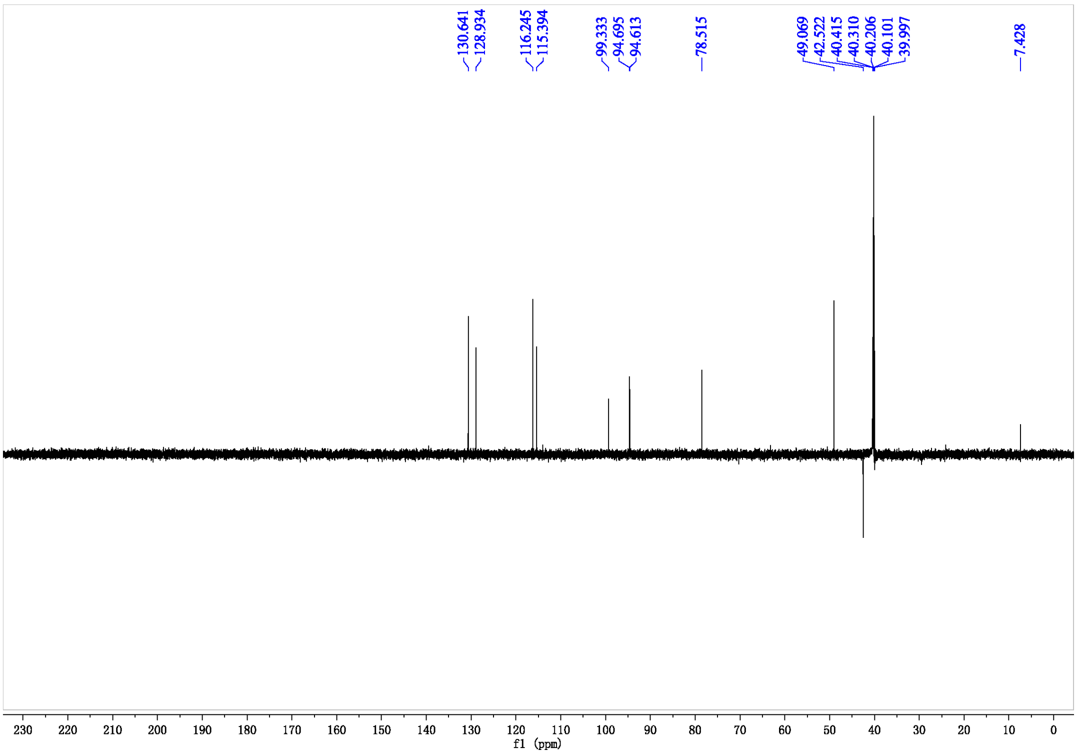

Figure S25. DEPT 135 spectrum (125 MHz) of **4** in DMSO-d_6

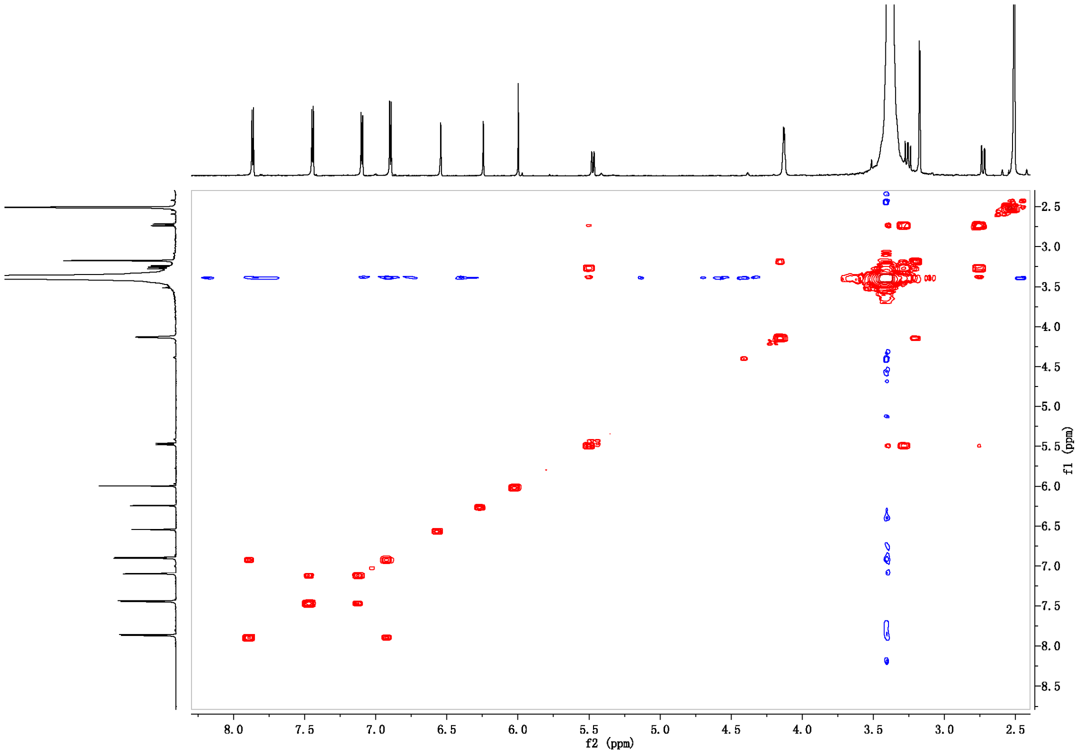

Figure S26. ^{1}H-^{1}H COSY spectrum of **4** in DMSO-d_6

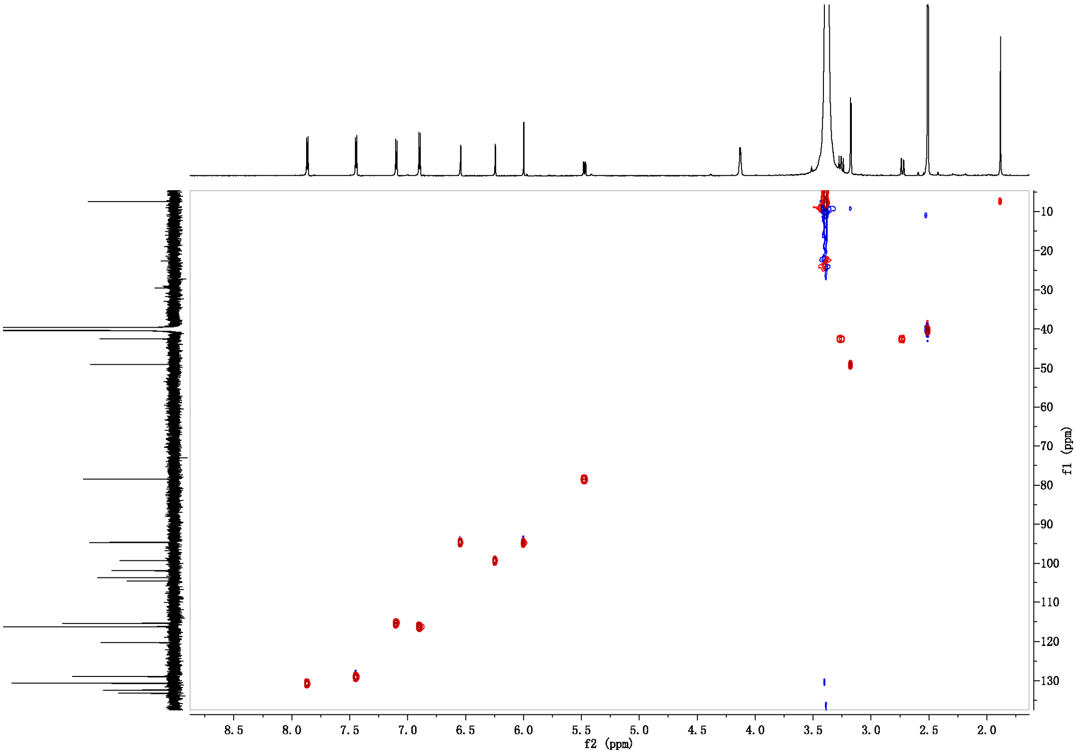

Figure S27. HSQC spectrum of **4** in DMSO-d_6

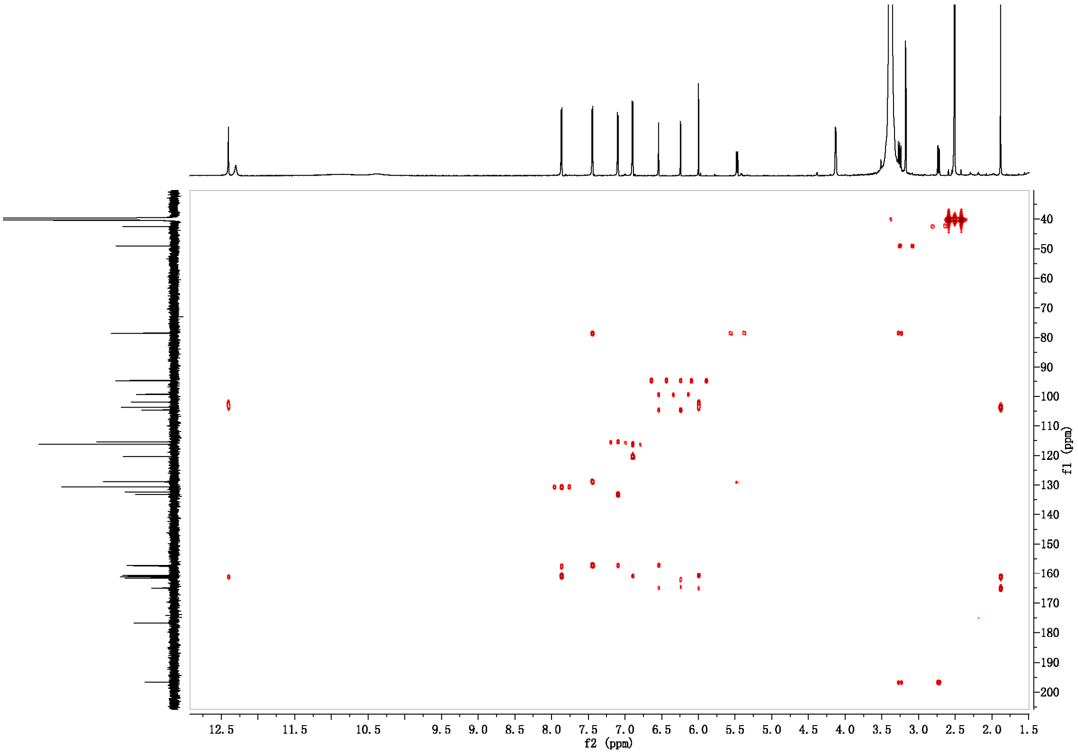

Figure S28. HMBC spectrum of **4** in DMSO-d_6

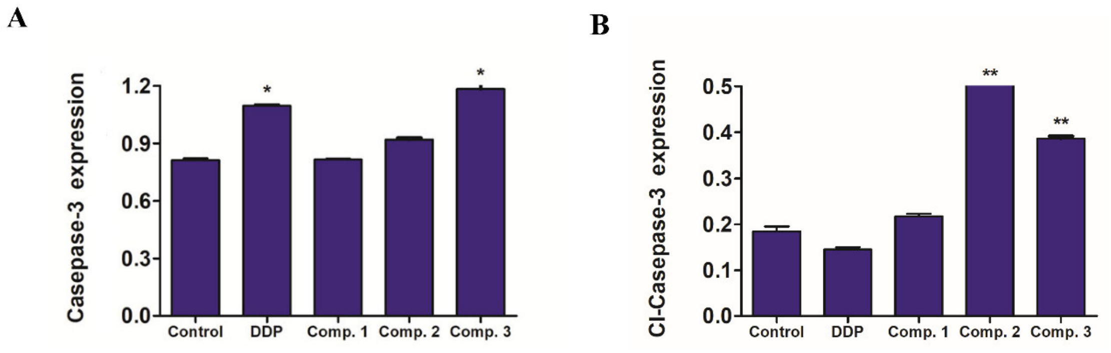

Figure S29. Effects of compounds **1-3** on expression of caspase-3 and cleaved caspase-3. A549 cells were treated with vehicle or the indicated compound for 24 h, and the relative levels of caspase-3 and cleaved caspase-3 were determined by western blot assays using *β*-actin as a control. (A) Quantitative analysis of caspase-3. (B) Quantitative analysis of cleaved caspase-3. Data are expressed as means from three independent experiments. *$P < 0.05$, **$P < 0.01$ vs control group.
